# Continuous-Flow Synthesis of Antiepileptic Drugs: Process Intensification, Sustainability Metrics, and Scalable Manufacturing

**DOI:** 10.1021/acsomega.6c04439

**Published:** 2026-08-25

**Authors:** Amit Upadhyay, Alka Raj Pandey, Ashwini Mishra, Shashank Tewari

**Affiliations:** † Faculty of Pharmaceutical Sciences, 720979 Mahayogi Gorakhnath University Gorakhpur, Arogya Dham, Sonbarsa, Gorakhpur, Uttar Pradesh 273007, India; ‡ 30082 Lloyd Institute of Management & Technology, Plot No. 11, Knowledge Park-II, Greater Noida, Uttar Pradesh 201306, India; § Center for Synthesis & Chemical Biology, School of Chemistry, 8797 University College Dublin, Belfield, Dublin D04 V1W8, Ireland; ∥ Department of Pharmaceutical Chemistry, Manipal College of Pharmaceutical Sciences, 76808 Manipal Academy of Higher Education, Manipal, Karnataka 576104, India

## Abstract

The current trend in antiepileptic drug (AED) development entails finding and creating safer, more effective technologies as the world continues to source AEDs, thereby creating a larger carbon footprint through the batch-process method of manufacturing AEDs, along with potential resource limitations and/or supply chain constraints on batch-manufactured products. This review will evaluate the change in AED manufacturing from the batch-process method to the continuous-flow process and the advantages associated with integrating environmentally friendly chemical methods into continuous-flow manufacturing processes to create AEDs and the new generation of AEDs with reduced waste. The review will examine examples of recent advances in continuous-flow processing of major molecules, including pregabalin, levetiracetam, lacosamide, and brivaracetam. In addition, the review will discuss the obstacles preventing a complete transition to an automated, environmentally friendly, and continuous-flow process for producing AEDs.

## Graphical abstract

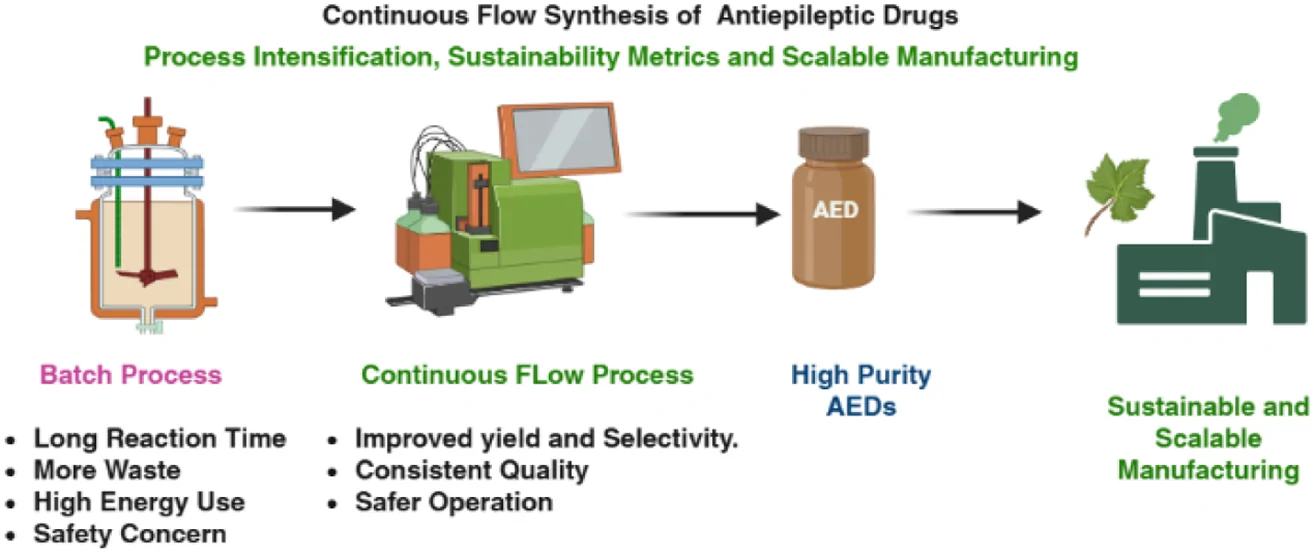

## 1 Introduction

Antiseizure medications, referred to as antiepileptic drugs (AEDs) or anticonvulsants, are a group of medications used to treat epilepsy and manage seizures. AEDs like pregabalin, levetiracetam, brivaracetam, diazepam, and sodium valproate are commonly used to manage epilepsy because of their broad spectrum of activity and relatively good safety record. These drugs work by changing the activity of neurons in the brain, reducing seizures caused by excessive electrical activity in neurons.^1^ Several different factors contribute to the growth of the global market for these drugs.^2^ The largest of these factors is the increasing number of people diagnosed with epilepsy throughout the world, as well as continual improvements in the diagnosis and treatment of epilepsy. At the same time, the introduction of newer AEDs that work better and have fewer side effects has increased the options for treating epilepsy and is driving the market’s expansion. Additional factors that contribute to the growth of the market are an improvement in public and professional awareness about epilepsy; increased access to health care; and an overall increase in healthcare spending. In addition to treating epilepsy, AEDs are also used to treat other neurological and psychiatric disorders, including the prevention of migraines, the treatment of neuropathic pain, and the treatment of bipolar disorder.^3^

Historically, the process of chemical manufacturing has been a batch process. Therefore, it had the associated risk of having large inventories of raw materials and taking too long to dissipate heat in the reactor because of the high volume of the batch, resulting in hot spots in the reactor and leading to poorly controlled products, large amounts of wasted material, and increased pollutant emissions to the environment.^4^ The limitations of the batch reactor method become critical for the production of complex AEDs. An innovative substitute for the batch process is continuous-flow processing (also known as flow chemistry) that uses narrow channels, tubular coils, or structured reactors for the uninterrupted feeding, mixing, and removal of reactants.^5^ Continuous-flow processing has established its place in the production of pharmaceuticals and fine chemicals.^6,7^ Compared with the limitations of batch processes, continuous processing offers many advantages. Continuous-flow processing allows for faster and more efficient heat and mass transfer rates through increased reaction rates, controlled residence times, and safer application of high-energy reagents under intensified reaction conditions (i.e., very high temperatures and pressures), thus providing access to an increasing number of reaction pathways than are possible with batch reactors.^4^ Furthermore, microreactors associated with continuous-flow processing enable the safe handling of extremely exothermic processes and hazardous intermediates within tightly regulated process windows.^8,9^ The environmental metrics employed to quantify sustainability improvements, such as the E-factor, process mass intensity (PMI), atom economy, and life cycle assessment (LCA), consistently demonstrate the superiority of well-designed continuous processes.^10^ The pharmaceutical sector stands to benefit substantially from flow technologies that minimize solvent waste, enable telescoped multistep sequences, and facilitate solvent recycling. Moreover, the linear scalability of flow processes from laboratory to production scale ensures that sustainability gains achieved during development are preserved upon industrial implementation, a critical consideration given that environmental impact scales with production volume.

Recent developments in continuous-flow synthesis have produced tremendous enhancements in many aspects. For example, the revolutionary potential of flow chemistry is demonstrated by the selective fluorination method used to synthesize flucytosine.^11^ Similarly, by combining continuous-flow synthesis, computational calculations, and solvent minimization, such approaches have dramatically lowered the E-factor of previously described pathways. Additionally, many pharmaceutical companies are investing in research and development into novel methods for drug synthesis using continuous-flow methods. For instance, Eli Lilly developed an efficient synthesis of evacetrapib using high-pressure hydrogenation processes in a continuous-flow setup.^12^ These examples not only show environmental advantages but also strong economic advantages such as lower capital expenditures, smaller equipment footprints, and the ability to produce on demand avoiding overproduction and storing large quantities of products for extended periods. Moreover, continuous processing was identified as the “highest priority” for advancing the field of green and sustainable manufacturing by the Pharmaceutical Roundtable of the American Chemical Society Green Chemistry Institute in its report published in 2007.^13^ Additionally, the growing acceptance of continuous-flow processing by regulatory agencies, such as the United States Food and Drug Administration and the International Council for Harmonization (ICH), has helped the pharmaceutical industry adopt continuous-flow technology for the synthesis of drug molecules.^14,15^

With respect to AEDs, continuous manufacturing offers an opportunity to address long-standing challenges in drug quality, affordability, and availability. Continuous-flow synthesis, integrated downstream processing, and real-time release testing, among other emerging technologies, have the potential to transform the manufacture of antiseizure medications by creating a more consistent means of drug delivery and supporting the advancement and development of next-generation therapies. This review focuses on the application of continuous-flow synthesis in the production of key AEDs, including brivaracetam, rufinamide, levetiracetam, pregabalin, sodium valproate, and diazepam. It examines recent advances in reaction design, catalytic strategies, process integration, and scale-up. This review focuses on the intersection of flow chemistry and AED synthesis, aiming to shed light on how advanced manufacturing technologies can facilitate the more sustainable, efficient, and scalable production of crucial neurological therapies. Earlier review articles have examined flow chemistry for either general active pharmaceutical ingredient (API) manufacture or pharmaceutical synthesis.^16,17^ Nonetheless, this is the first report to specifically address the integration of continuous-flow synthesis of AEDs with process intensification, sustainability metrics, reactor engineering, process scale-up, and industrial translation.

## 2 Fundamentals of Green and Scalable Flow Chemistry

A continuous-flow synthesis setup consists of a modular, integrated machine system made up of four main functional areas: fluid delivery, mixing/reaction, downstream processing, and analytical monitoring (Figure 1).^18^ Liquid reagents and gases are supplied at fixed flow rates from high-precision piston or syringe pumps and mass flow controllers in the feed section, which is the starting point of the process. These streams then merge within the mixing units (e.g., T-junctions or micromixers) before being fed into the reactor zone, typically consisting of temperature-controlled coils, chip-based microreactors, or packed-bed columns employing solid-supported catalysts.^19^ These units allow for the precise control of residence time and heat exchange, often operating under elevated pressures supplied by back-pressure regulators, enabling the use of superheated solvents. Also included in the system are workup and purification modules, including membrane-based phase-separation devices and scavenger columns, to perform liquid–liquid extractions and remove impurities in real time, as well as to manage the reaction itself. A defining feature of modern systems is the addition of Process Analytical Technology (PAT), which incorporates inline IR, NMR, and UV–vis spectrometers.^9,20^ The PAT provides continuous data streams that can be used to create automated feedback loops within the system, enabling the automatic adjustment of process parameters such as flow rate or temperature to improve yield and selectivity.

**Figure 1:**
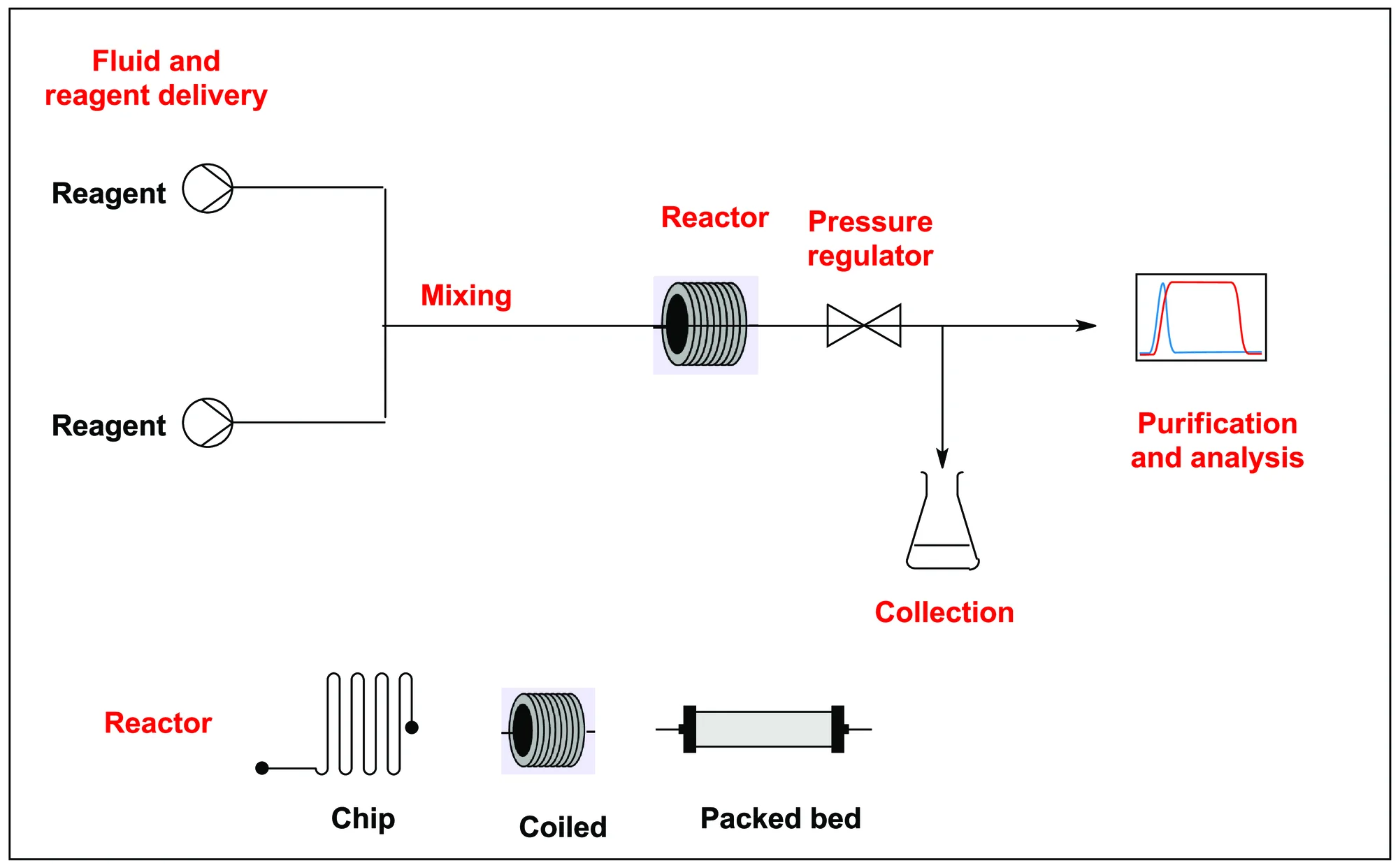
Components of a basic continuous-flow processing system.

Flow chemistry is a field within synthetic organic chemistry that employs a continuous flow of various reagents, introduced through pumps and combined within a continuous reactor, such as a plug-flow reactor (PFR) or a continuous stirred-tank reactor (CSTR).^21,22^ In contrast to traditional batch processing, which is typically done in round-bottom flasks, flow chemistry provides numerous advantages, including improved mass and heat transfer, enhanced safety, greater reaction efficiency, minimized waste, better scalability, and improved consistency.^23^ As a result, flow chemistry facilitates the precise management of reaction conditions and allows for real-time tracking and analysis of reaction kinetics, leading to high-quality outputs and more efficient processes. In addition, the increase in both heat and mass transfer also gives operators more control over the temperatures and concentrations within their systems. This new technology platform represents an unprecedented solution for achieving the 12 principles of green chemistry by allowing for process intensification through waste minimization and by improving energy efficiency.^24^ Because of the many unique properties of flow chemistry, including a high surface-area-to-volume ratio, fast mixing, and the ability to maintain good thermal control during both exothermic and potentially explosive operations, it significantly reduces environmental impact while simultaneously increasing the efficiency of chemical reactions.^25^ Flow chemistry has created a bridge between developing chemical reactions in the laboratory and producing them at an industrial scale by providing reproducible, scalable, and inherently safer ways of conducting reactions.^26^ These advantages have contributed to an increase in the adoption of flow-based chemistry by both academia and numerous industries, especially in the pharmaceutical, fine chemicals, and materials science industries.^27^

Flow chemistry offers numerous benefits for large-scale production compared to conventional batch chemistry, including the ability to more easily translate lab-scale reactions into larger production formats without compromising reaction efficiency (Figure 2).^28,29^ There are two primary strategies for scaling up reactions in flow chemistry: numbering up and sizing up.^30^ Numbering up refers to increasing the number of channels within a microreactor, while sizing up involves extending the length and/or diameter of these channels. In addition to passive microreactors, which rely on pump-delivered flow energy for mixing, active microreactors that employ external energy sources are frequently used, especially for scaling up heterogeneous reactions. Various types of active continuous reactors have been documented, including rotating disk reactors, oscillatory flow reactors, thin-film rotating reactors, continuous stirred-tank reactors (CSTRs), and ultrasonic reactors.^31–33,34^ For instance, effective and smooth scale-up of the aerobic oxidation processes for primary alcohols and aliphatic aldehydes has been achieved in a compact heat exchanger reactor, while maintaining the segmented flow characteristics and intensified conditions observed in laboratory experiments. Utilizing a Kobelco SMCR with a reaction volume of 43 mL, Guicheret and colleagues achieved a productivity rate of 20 g h^–1^ for the aerobic oxidation of benzyl alcohol to benzaldehyde and 692 g h^–1^ for the conversion of 2-ethylhexanal to 2-ethylhexanoic acid.^35^ These findings significantly highlight the potential application of milli-structured reactors in pharmaceutical process chemistry. Additionally, scientists are starting to employ digital process-development workflows that utilize mathematical models developed by taking much data from lab testing and pilot testing. These models are used to provide scientists with a pathway in an *in silico* environment for determining and defining the design space across multiple parameters and process variables, thereby eliminating hundreds of physical tests used in classical methods of process development. These developments have established pathways to ensure sustainability benefits observed in bench-scale tests remain valid when scaled up to commercial production. Continuous-flow synthesis is a sustainable methodology that takes advantage of process intensification to perform reactions with greater accuracy than batch methods (Figure 3).^36^ A key benefit of the microreactor-based system is the absence of temperature gradients or “hotspots”,enabling higher selectivity and less byproduct formation.^37^ High surface-area-to-volume-ratio microchannels enable higher reagent concentrations and improve reaction rates through techniques such as the safe manipulation of superheated solvents, which substantially decreases the volume of hazardous solvents in the process, resulting in reduced waste generation.^38^ Flow platforms provide a straightforward means to perform Life Cycle Assessment (LCA) integration.^39^ The modular, telescoped nature of flow chemistry allows multiple reactions, extraction, and purification steps to be executed in continuous processing. As a result, the carbon footprint and aquatic toxicity can be quantified and reduced in real time, in line with the principles of the circular economy.^40^ Recyclable starting materials and solvents can be seamlessly reused. The green benefits of flow methodology can be tracked and realized throughout the entire life cycle of a molecule, rather than simply during the reaction process. Finally, safety and hazard mitigation are also major advantages of flow chemistry. In traditional batch chemistry, a cooling failure could result in the total destruction of a vat in an uncontrolled explosion, however, in flow processing the “hold-up” (i.e., the amount of material in the system at any one time) is just a few hundred milliliters at most and can be safely dissipated via the effluent.^41^ Furthermore, volatile intermediates can be contained safely within the flow equipment, which is usually not possible in batch synthesis. This allows continuous-flow processing to be a safer and more sustainable choice for modern synthesis.^42,43^

**Figure 2:**
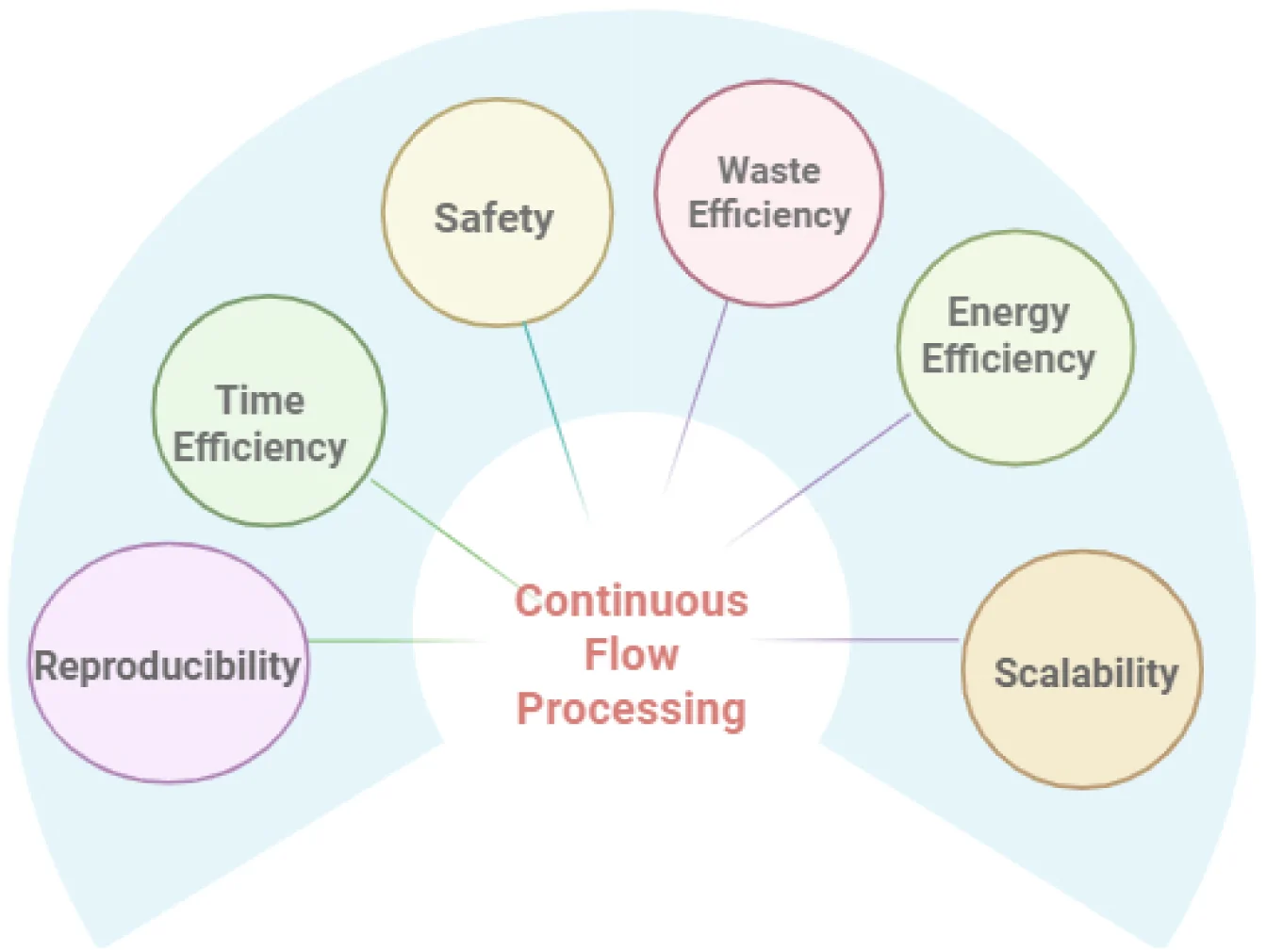
Advantages of a continuous-flow processing system.

**Figure 3:**
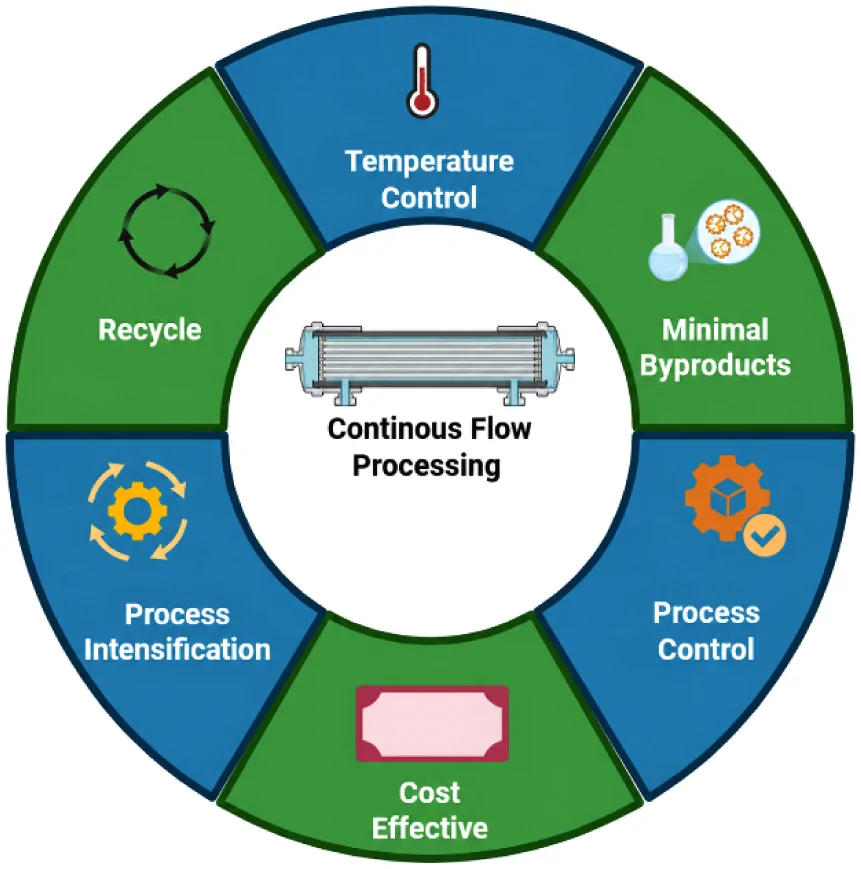
Sustainability metrics for continuous-flow processing.

To facilitate comparison among the many continuous-flow synthetic methods described in detail in the sections that follow, Table 1 summarizes the primary features of continuous-flow syntheses of reported AEDs. Table 1 includes the reactor configuration, the major chemical transformation, the performance of the chemical reaction under continuous-flow conditions, the processing time, any reported green metrics, and the most significant improvements resulting from the use of continuous-flow processing. The summary provides a useful resource for comparing methods before discussing each case study in detail.

**Table 1:** Comparative Summary of Reported Continuous-Flow Syntheses of AEDs Presented in This Review

Drug	Reactor configuration	Key flow strategy	Overall yield reported/Residence time	Key achievements from continuous-flow processing
**Pregabalin**	Packed bed reactor	A two-step telescoped asymmetric Michael addition is followed by catalytic nitro reduction.	89% γ-lactam intermediate/ 3.5 h.	Telescoped synthesis is performed without intermediate isolation using heterogeneous catalysis, achieving 87% ee.
**Pregabalin**	Packed bed reactor	Asymmetric conjugate addition is conducted using an immobilized nickel–bisdiamine catalyst.	90% for the intermediate. Continuous operation is maintained for more than 5 days	The process demonstrates stable long-term continuous operation with negligible catalyst leaching and effective catalyst reuse.
**Pregabalin**	Continuous flow system	A three-step heterogeneous catalytic synthesis	67%	Three reaction steps are performed sequentially without intermediate isolation.
**Sodium Valproate**	Continuous flow system	Dipropylation, hydrolysis, and salification processes	87%/ 41 min	The process eliminates corrosive acid-mediated decarboxylation, incorporates inline extraction, and achieves product purity greater than 99%. E-factor = 10.8; PMI = 11.8.
**Diazepam**	Continuous flow system	N-acylation followed by substitution or cyclization	55–96%/15 min	The optimized protocol enables rapid multistep synthesis, yielding isolated products with purity greater than 98%.E-factor reduced from 36 to 9.
**Brivaracetam**	Continuous flow system	Visible-light asymmetric photocatalytic addition	98% (photochemical intermediate); 44% overall yield/ 25 min	The reaction time decreased from 4 h in batch processing to 25 min under flow conditions, and gram-scale synthesis was achieved.
**Levetiracetam**	Continuous photoflow reactor	Organophotocatalytic α-functionalization and subsequent multistep continuous synthesis	24%/30–60 min	Continuous multistep photochemical synthesis approach for levetiracetam production.
**Rufinamide**	Copper tubing reactor	Copper catalyzed cycloaddition	92%/ 11 min	In situ generation and rapid consumption of azide intermediates under continuous-flow conditions.
**Rufinamide precursor**	Five-stage integrated microreactor network	Multistep synthesis with inline liquid–liquid separations	82%/95 min	The process integrates three reaction steps with two inline separation units, operating in a continuous manner.
**Rufinamide**	One-flow continuous-flow platform	Integrated azidation and CuAAC using a single solvent system	90%	Single-solvent continuous process without intermediate purification.
**Rufinamide**	Supercritical CO_2_ flow reactor	Continuous synthesis with inline chromatographic purification	52%	Continuous synthesis integrated with inline chromatographic purification.

## 3 Continuous Manufacturing of AEDs: Case Studies

### 3.1 Pregabalin

Pregabalin is a significant member of the GABA-derivative drug family. It serves as both an anxiolytic and an anticonvulsant and is used in the treatment of epilepsy, neuropathic pain, fibromyalgia, and generalized anxiety disorder.^44,45^ A number of reported continuous-flow processes for the manufacture of pregabalin involve similar fundamental reactions and differ only in the specific catalyst employed, reactor design, or operating conditions. The studies have therefore been classified into three general categories: 1) asymmetric Michael addition routes; 2) catalytic hydrogenation of nitro intermediates; and 3) integrated, telescoped, continuous-flow manufacturing. For instance, Kobayashi and colleagues created a novel composite by applying a chiral nickel–diamine complex through wet impregnation onto a mesoporous aluminosilicate material known as MCM-41.^46^ The prepared catalyst exhibited remarkable enantioselectivity in the conjugate addition of dimethyl malonate to the corresponding aliphatic nitroalkene in a continuous-flow packed-bed system. The resulting chiral product **1** was further converted into γ-lactam **2** via nitro reduction using hydrogen gas and Pd/DMPSi-C as a heterogeneous catalyst. Compound **2** acts as a chiral precursor for (*R*)-pregabalin. In a 3.5-h run, the two-step telescoped flow method yielded 186 mg (89% yield) of compound **2** with an enantiomeric excess (ee) of 87% (Scheme 1). The heterogeneous catalyst employed in this research depended on noncovalent interactions for the immobilization of the chiral ligand, which resulted in leaching issues and limited reusability.

**Scheme 1:**
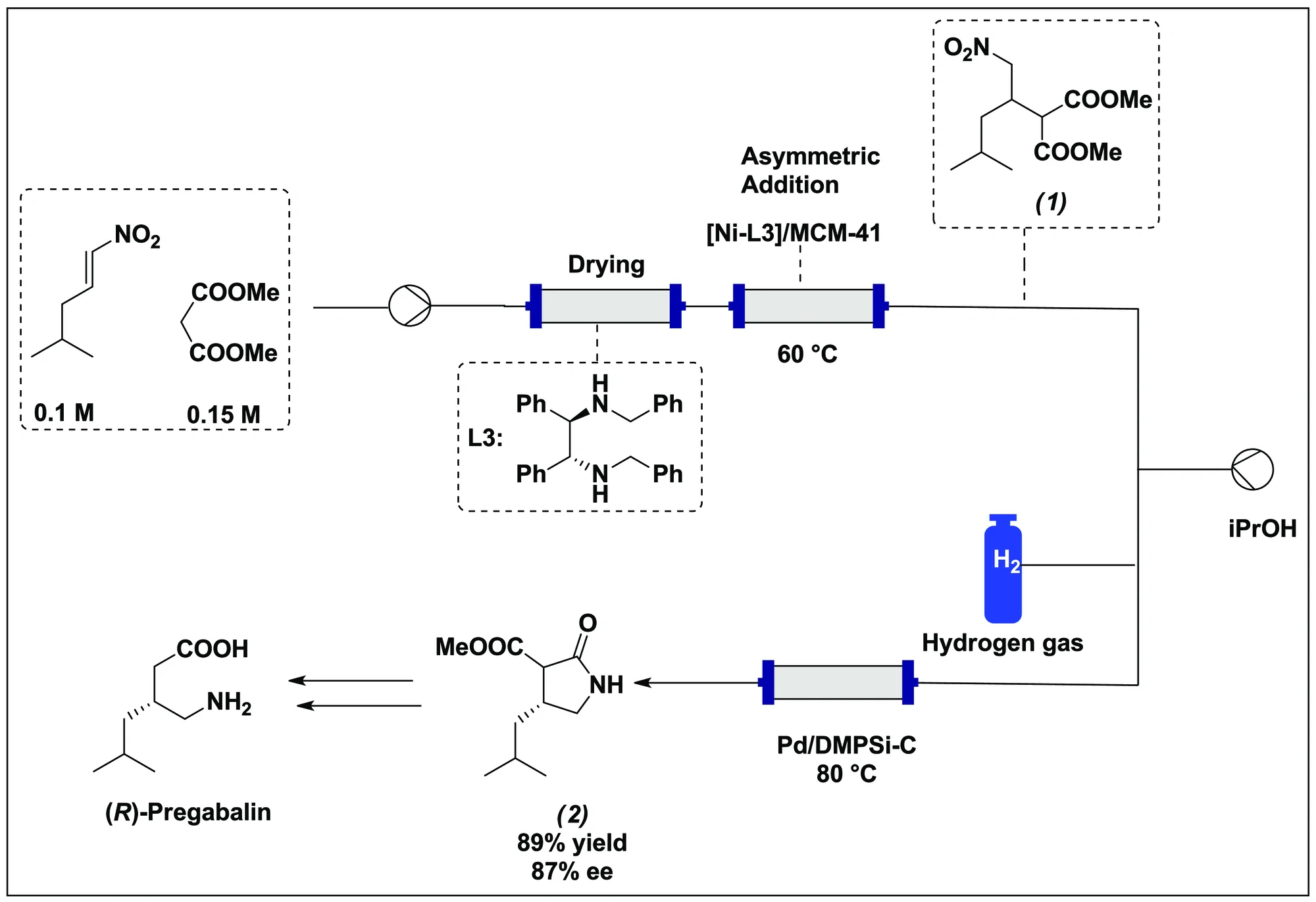
Telescoped Continuous Synthesis of (*R*)-Pregabalin

The work of Kramer et al. and their method for performing continuous pregabalin synthesis via a packed-bed continuous-flow approach represent major advances in this field.^47^ In this study, a chiral nickel bisdiamine complex was covalently bound to a porous organic polymer (POP), thereby reducing metal leaching while maintaining excellent catalytic efficiency (Scheme 2). The POP catalyst was incorporated into a packed-bed reactor for the continuous production of a chiral pregabalin intermediate (**1**) via asymmetric conjugate addition. The reactor has operated continuously for more than 5 days, yielding more than 4 g of the chiral intermediate with 90% yield and 90% ee. The study illustrates that improvements in process robustness, catalyst recyclability, and operating stability can be achieved through catalyst immobilization and reactor design, demonstrating that pharmaceutical manufacturing can practically benefit from the use of continuous-flow asymmetric catalysis.

**Scheme 2:**
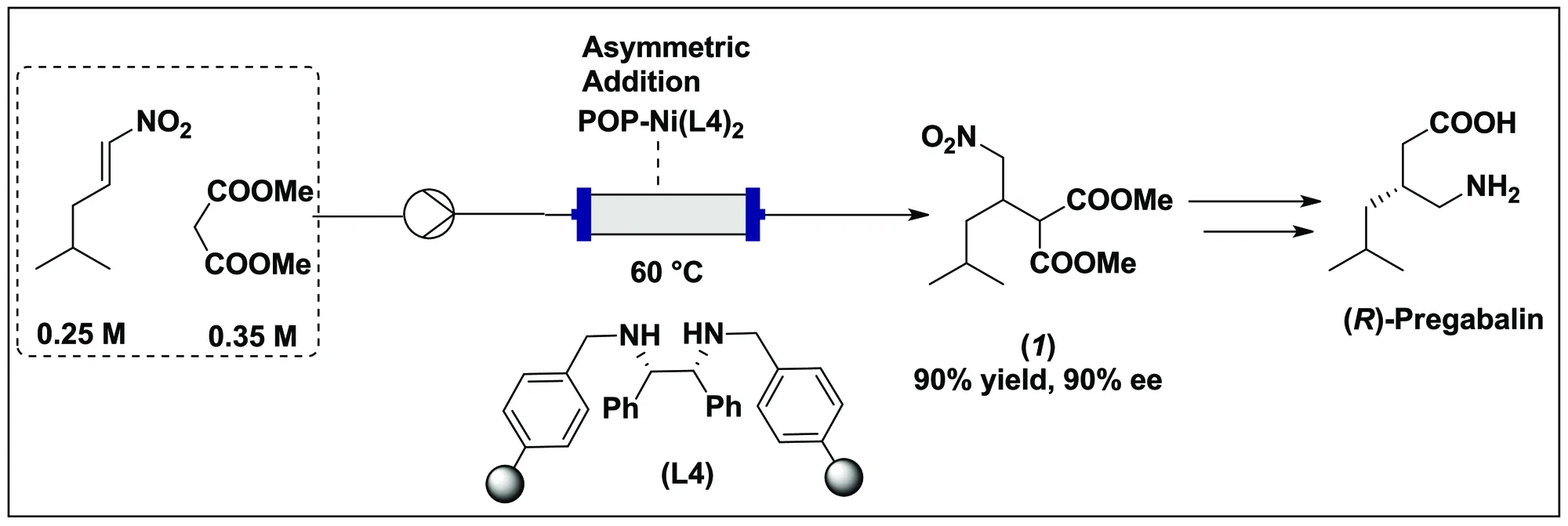
Asymmetric Synthesis of (*R*)-Pregabalin Using Continuous Flow Processing

Kobayashi and colleagues also synthesized the racemic variant of the pregabalin precursor **2** through a three-step, telescoped, continuous-flow process involving a Knoevenagel condensation (Scheme 3).^48^ Following the distillation of the reaction solution from the three-step, sequential-flow process, compound **2** crystallized as a white solid after recrystallization. By heating **2** in hydrochloric acid, followed by neutralization, transformed it into pregabalin. The final product was obtained in 67% yield based on methyl malonate. Additionally, Seeberger and his team described a multistep flow assembly setup featuring interchangeable reaction modules for producing racemic pregabalin, phenibut, baclofen, and rolipram.^49^

**Scheme 3:**
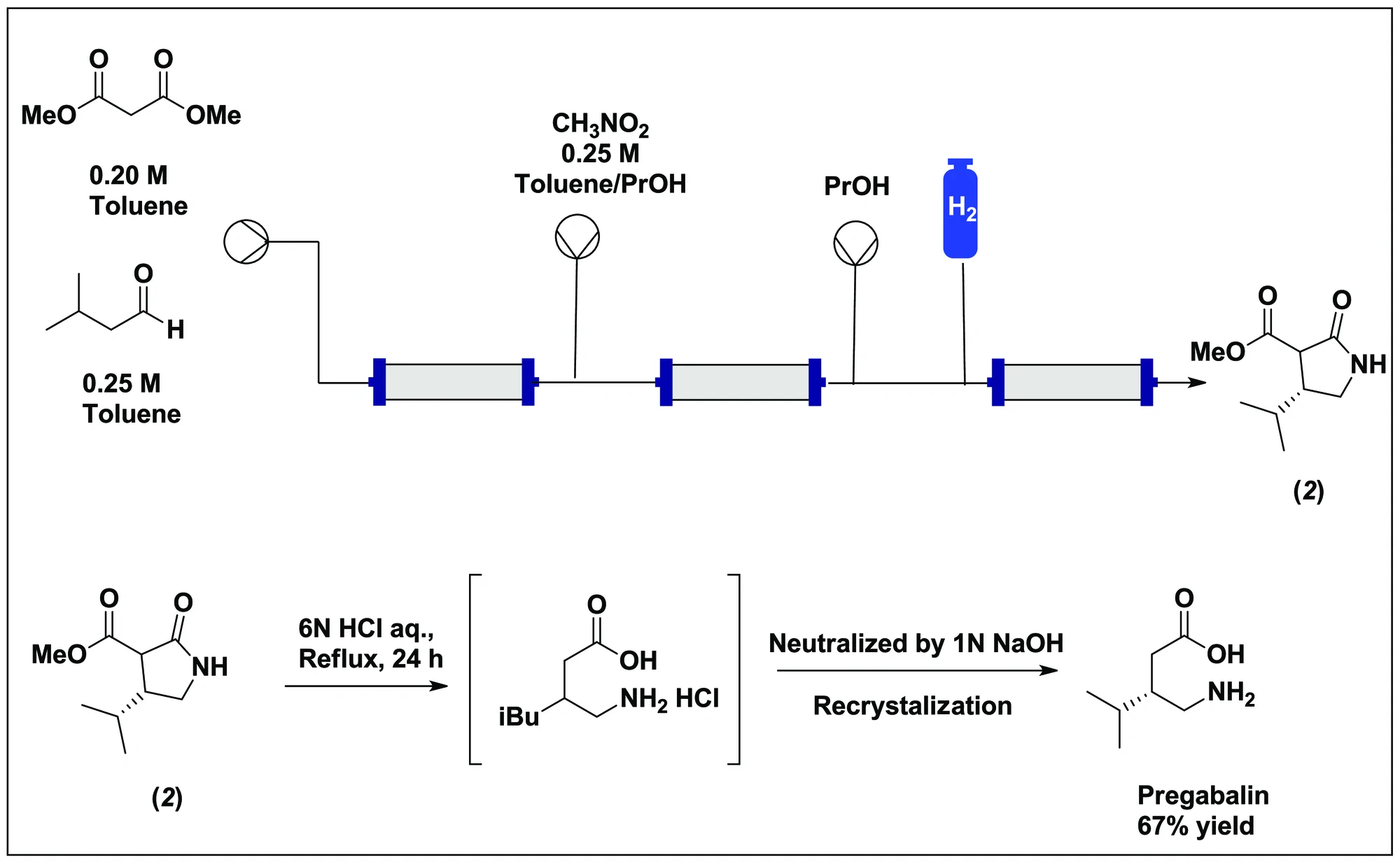
Telescoped Multistep Continuous-Flow Strategy for Synthesis of (*R*)-Pregabalin

Of all the techniques discussed to date , the most commonly used method for obtaining the requisite chiral γ-amino acid framework of pregabalin has been the asymmetric Michael addition, due to its superior stereocontrol and the ease with which it creates chiral γ-amino acids.^50^ Later studies have shown that the principles of this basic reaction can also be utilized to enhance other variables, such as catalytic activity, through immobilization or homogeneous catalyst use; overall productivity, by packing reactors to increase reactor volume; and operational stability, through heterogeneous catalytic systems. Overall, the most significant advancements in this time frame can be attributed to process intensification and advances in catalyst engineering.

### 3.2 Sodium Valproate

Sodium valproate **3**, the sodium derivative of valproic acid, is extensively utilized for the treatment of both generalized and partial seizures in patients, particularly in cases of epilepsy and bipolar disorder, as well as for migraine prevention.^51^ Sodium valproate is only available by prescription and is listed on the World Health Organization’s List of Essential Medicines.^52^ Valproic acid is a branched, short-chain fatty acid derived from valeric acid and was first synthesized by the American chemist Burton in 1882.^53^ The typical industrial batch production of sodium valproate generally commences with either ethyl cyanoacetate or diethyl malonate; these undergo a reaction with propyl bromide facilitated by an alkoxide and a phase-transfer catalyst.^54,55^ However, these processes are associated with significant disadvantages, including lengthy synthetic procedures, extended reaction durations, repeated use of sodium hydroxide (NaOH), the requirement for expensive corrosion-resistant equipment for high-temperature, acid-catalyzed decarboxylation, and substantial production of inorganic salt waste.

In 2025, Wan and colleagues developed a simplified three-step flow process with the goal of creating a more effective and sustainable continuous synthesis technique for sodium valproate.^56^ The procedure included a simple method that did not rely on corrosive, acid-mediated decarboxylation. A dipropylation reaction using propyl chloride was performed, and successive basic hydrolysis and salification afforded sodium valproate **3** with a total yield of 87% and a purity of over 99% after an inline extraction to eliminate impurities (Scheme 4). This was accomplished with just 41 min of residence time, yielding a throughput of 552 g/day. With an E-factor of 10.8 and a process mass intensity of 11.8, this method’s green metrics were far lower than those of the current batch-production technique, indicating a more sustainable strategy.

**Scheme 4:**
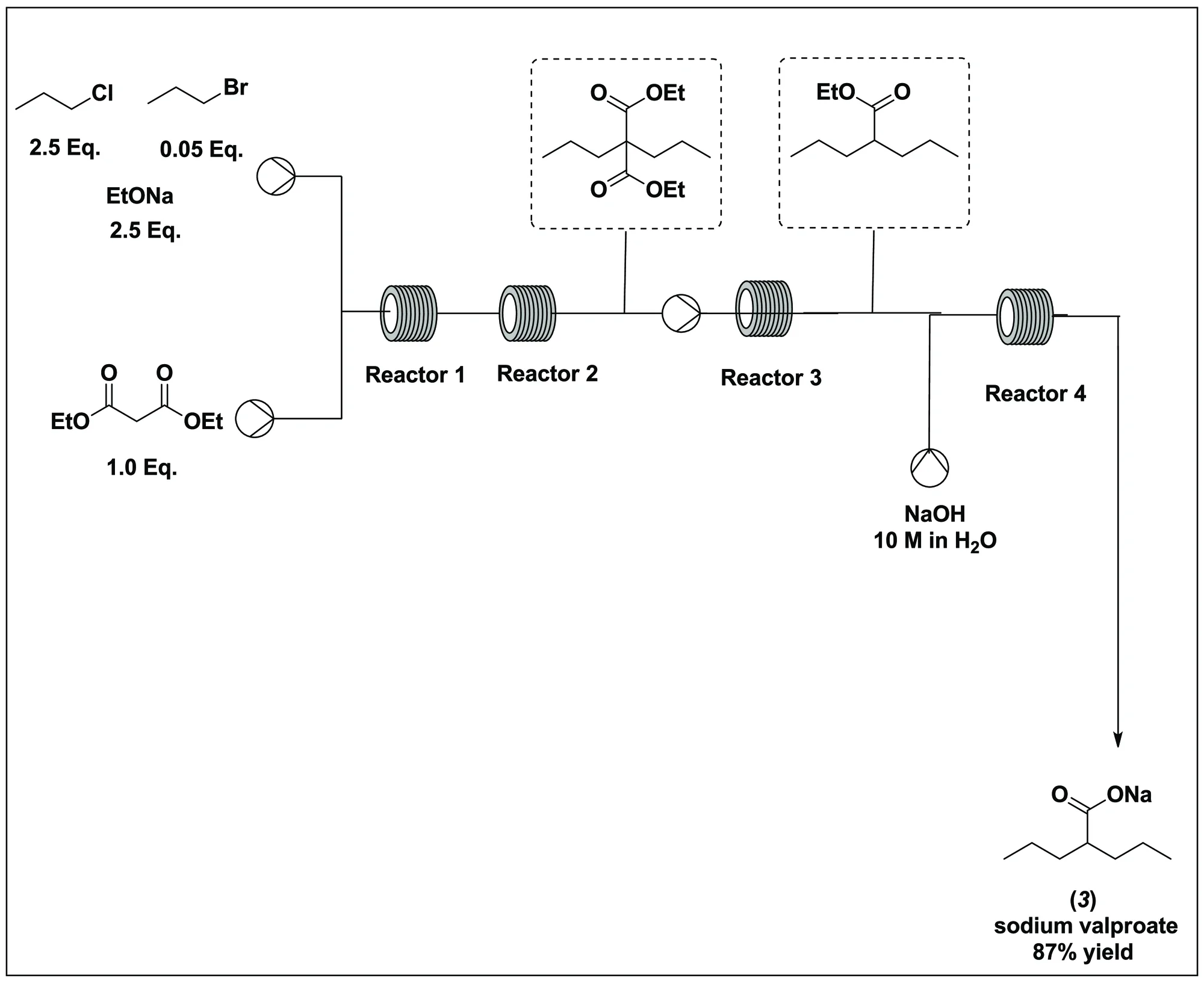
Continuous-Flow Synthesis of Sodium Valproate

### 3.3 Diazepam

Diazepam **4**, a rapidly acting antianxiety medication, is deemed an essential drug by the World Health Organization.^52^ Unfortunately, diazepam is highly prone to shortages, which can lead to increased prices and heightened demand for other benzodiazepines during periods of limited supply.^57^ Given the rising prescription rates, implementing a strong, scalable continuous manufacturing process for diazepam is timely and represents an important opportunity for process enhancement.^58,59^ Several studies outline the continuous production of diazepam through a two-step process involving *N*-acylation, followed by sequential substitution and cyclization reactions, commencing with 5-chloro-2-(methylamino)­benzophenone (Scheme 5).

**Scheme 5:**
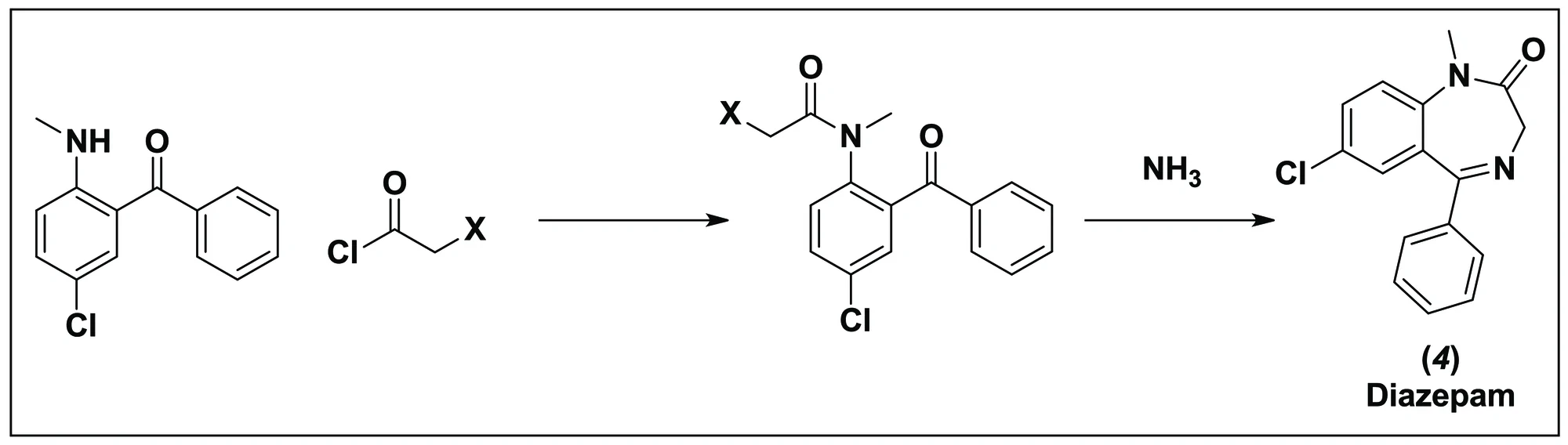
Two-Step Synthesis of Diazepam

Adamo et al. developed a procedure to synthesize diazepam by solubilizing their reactants in *N*-methyl-2-pyrrolidone (NMP), which resulted in a 78% crude yield (94% yield based on HPLC analysis) (Table 2).^60^ Ewan et al. published their results on high-throughput experimentation to optimize diazepam synthesis conditions.^61^ They obtained diazepam (70% purity) using a microfluidic reactor system with toluene and methanol. This technique was also used by Rogers et al. in a subsequent publication, where they reported processing 2800 dose equivalents of diazepam.^62^ Collins et al. used ammonium acetate (NH_4_OAc) and developed a fully automated system (AutoSyn) to produce diazepam (yield 96%, purity 81%).^63^ Bédard et al. aimed to minimize waste in their flow process to improve the E-factor.^64^ To eliminate the need for a separate extraction solvent, a solvent suitable for both reaction and extraction was investigated and 2-methyltetrahydrofuran (2-MeTHF) was selected. The solubility of starting materials and most products in 2-MeTHF was greater than 0.9 M, and its low density of 0.85 g/mL reduced the mass of waste generated. Similarly, 2-MeTHF remained unreactive with reagents at elevated temperatures and is immiscible with water, which enables inline aqueous extraction without the need for additional solvent. Aqueous ammonium hydroxide (NH_4_OH) was selected as the ammonia source to avoid introducing excess organic solvent. Additionally, the use of an aqueous stream was necessary to prevent clogging caused by the formation of ammonium chloride. They achieved only a 55% yield but were able to reduce the E-factor of the process originally developed by Adamo et al. from 36 to 9.

**Table 2:**
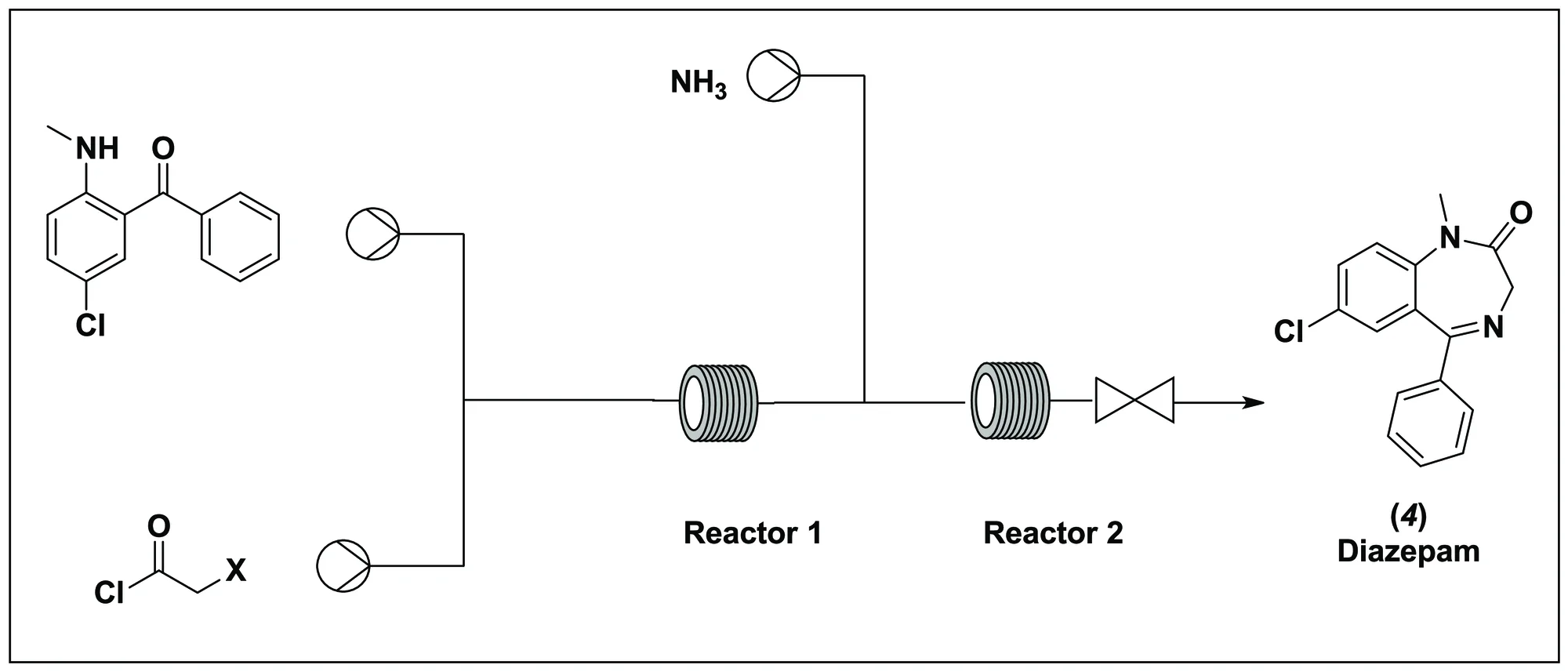
Comparison of Diazepam Synthesis under Different Conditions Using Continuous-Flow Processing

S. No	X	Acetamide (M)	Acid chloride (eq )	Ammonia analogue	Solvents	Yield (4)	Ref.
1.	–Br	1	1.2	7 M NH_3_	NMP, MeOH, H_2_O	78%	^60^
2.	–Br	0.1	2	7 M NH_3_	Toluene, MeOH	100%	^61^
3.	–Br	1.0	1.2	7 M NH_3_	NMP, MeOH, H_2_O	79%	^62^
4.	–Cl	0.4	1.0	NH_4_OAc	Toluene, MeOH	96%	^63^
5.	–Cl	1.0	1.25	NH_4_OH	2-MeTHF, H_2_O	55%	^64^

Thompson and colleagues have recently presented a telescoped flow synthesis involving two microreactors arranged in series, maintained at temperatures of 0 and 60 °C (Scheme 6).^65^ They also found that saturated solutions of ammonium bromide (NH_4_Br) in 30% ammonium hydroxide (NH_4_OH) convert acetamide into diazepam at moderate temperatures without requiring a back-pressure regulator (BPR). The NH_4_Br/NH_4_OH blend generates ammonia on demand through the common-ion effect, thereby eliminating the need for a BPR, which could represent a potential process failure point. Through these efforts, diazepam was obtained in a yield of 96% with a purity of 91% in just 15 min. Following one recrystallization, diazepam with a purity exceeding 98% was obtained.

**Scheme 6:**
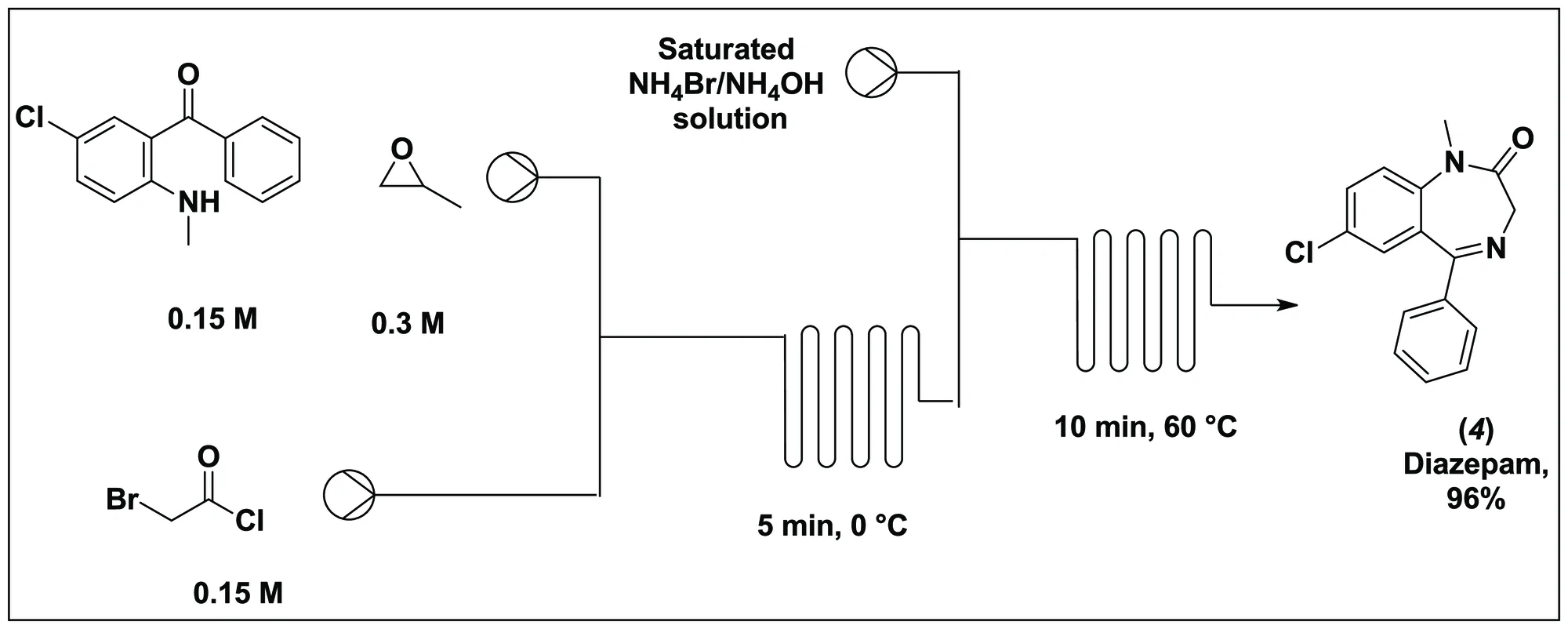
Strategy for Multi-Step Continuous-Flow Synthesis of Diazepam

Various methods reported for making diazepam by continuous-flow chemistry use different ammonia sources and solvent systems to optimize the two steps: the *N*-acylation step to form the haloacetamide intermediate and the cyclization step, which generates the benzodiazepine ring *via* intramolecular nucleophilic substitution by using an ammonia source. Although each process produced diazepam in good yield, different reaction media affected reagent compatibility, substrate solubility, reaction times, and ease of use. Most researchers used methanolic ammonia solutions as their ammonia source for diazepam synthesis , making it easy to maintain a homogeneous reaction medium during continuous-flow processing and enabling efficient formation of haloacetamides. Similarly, aqueous ammonia systems are a safer and easier way to use ammonia, since there is no need to handle concentrated ammonia solutions. Thus, solvent selection is a compromise between reaction performance, process safety, reagent handling, and downstream processing needs rather than a clear choice of a superior solvent for diazepam synthesis. These studies also demonstrate that careful choice of ammonia sources and solvent systems can improve the overall feasibility and robustness of continuous-flow methods for producing diazepam while still delivering high yields.

### 3.4 Brivaracetam

Brivaracetam is a new third-generation AED that has a specific affinity for synaptic vesicle protein 2A (SV2A).^66^ Brivaracetam reduces presynaptic neurotransmitter release by binding with SV2A.^67^ In the European Union, brivaracetam (BRV) is recommended as an adjunctive treatment for focal-onset (partial-onset) seizures (FOS) in patients older than 2 years.^68^ Brivaracetam (5) is structurally related to levetiracetam (6), but possesses an n-propyl substituent at the C4 position, which significantly enhances its affinity for the synaptic vesicle protein SV2A (Scheme 7).^69^ Brivaracetam’s synthesis requires a high degree of control over both of the stereogenic centers, since there are two stereogenic centers. Historically, the synthesis of brivaracetam involves a chiral resolution or a chromatographic separation between stereoisomers to obtain the product with the desired stereochemistry.^70^ These methods have historically been low yielding and used multiple steps due to low chiral selectivity. To address those issues, Franco and coauthors developed a new method of enantioselective synthesis via a visible-light-induced asymmetric Giese addition using a chiral rhodium photocatalyst (Δ-RhS) to add the key stereocenter to the product.^71^ In batch synthesis, the conjugate addition of an α-aminoalkyl radical generated from a Hantzsch ester to an α,β-unsaturated acylimidazole produced 7 in quantitative yield and an excellent enantiomeric ratio of 98:2 (er). In order to avoid issues with scale in photochemical reactions, the authors translated the batch photochemical process into a continuous-flow photoreactor system, where the continuous flow allowed for increased light penetration, improved mass transfer, and shorter reaction times than were achieved in batch. Under optimized conditions (40 °C, CFL irradiation, 1 mL min^–1^ flow rate), **7** was produced at 98% yield with 96:4 er after only 25 min of residence time compared with a 4 h residence time required in batch.

**Scheme 7:**
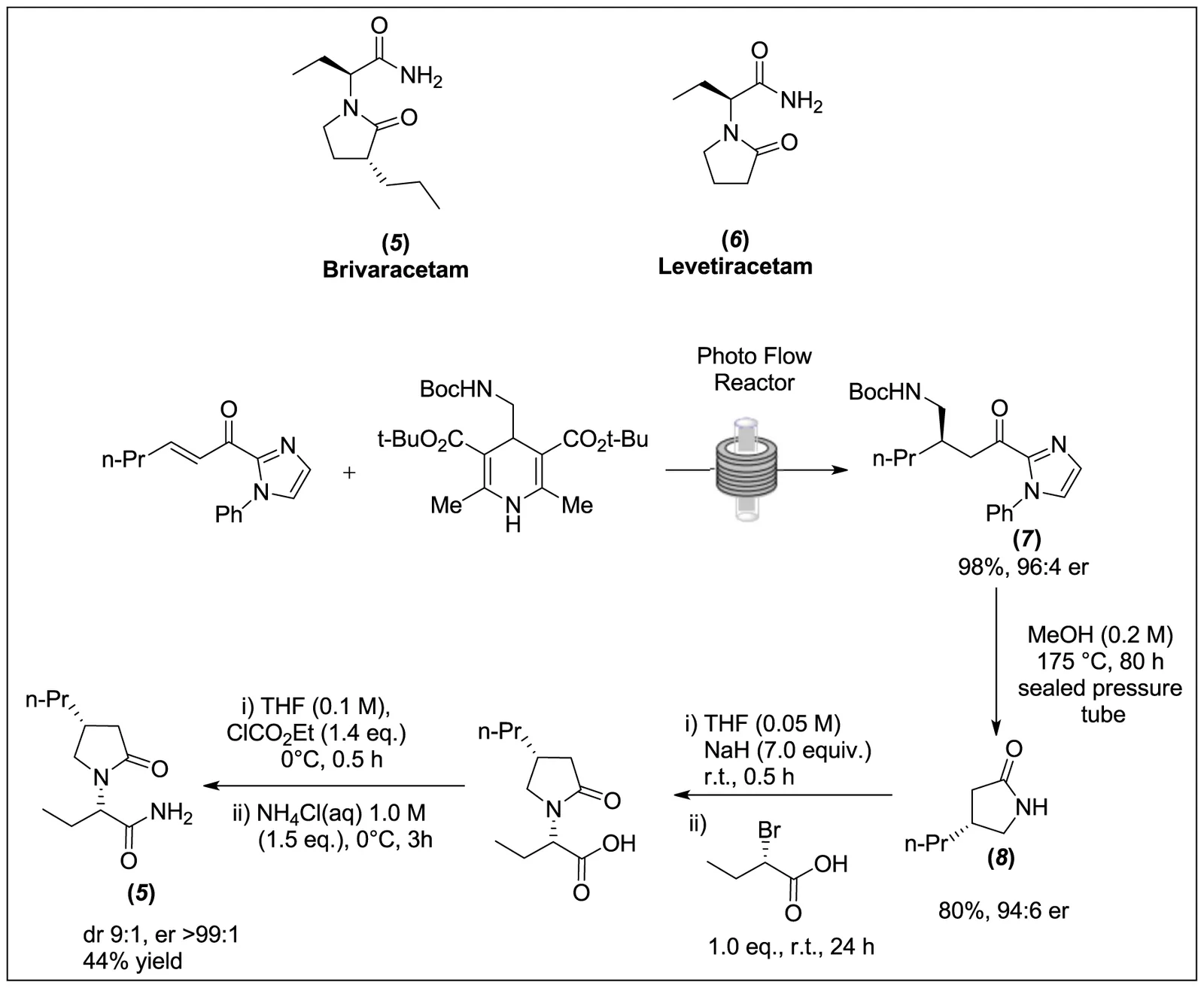
Asymmetric Photocatalysis Combined with Continuous-Flow Processing Strategy for Synthesis of Brivaracetam

The efficiency of the photoflow technique for synthesizing larger quantities of an intermediate compound was convincingly shown through the successful gram-scale synthesis of 1.7 g of intermediate **7** with a 94% yield in 75 min. Once the intermediate compound was obtained, it was converted into (*R*)-4-propylpyrrolidin-2-one (**8**) by methanolysis and intramolecular cyclization, ensuring no loss of stereochemical integrity. The final step, which produced the desired target API brivaracetam, was accomplished by *N*-alkylation with (*R*)-2-bromobutanoic acid followed by amidation; these two steps resulted in an overall yield of 44%, a 9:1 diastereomeric ratio, and an er of 99:1 for brivaracetam. The approach used in this work significantly reduced the amount of time needed to complete the reactions, improved the scalability of the photoflow method, and eliminated the need for labor-intensive methods to form chiral compounds. Overall, these results demonstrate how photoflow chemistry can be utilized in future manufacturing processes to produce an antiepileptic medication.

The difference in reaction time between continuous photoflow synthesis and batch synthesis of brivaracetam is pronounced, indicating that photoflow chemistry is faster than batch chemistry. However, practical trade-offs and limitations affect the application of continuous photoflow chemical processes relative to batch processes in general. The inherent design of continuous photoflow reactors allows for much higher photon utilization due to their very high surface area-to-volume ratio.^72^ They also provide more uniform interior illumination, better temperature control, and greater reliability in photochemical reactions compared with conventional batch processes; these advantages often result in shorter residence times, improved energy efficiency, and a safer operational environment due to reduced inventories of reactive intermediates and improved continuous heat removal during the reaction.

However, there are also practical challenges that accompany the benefits of continuous photoflow processing. For example, specialized reactor designs are often necessary for industrial use, with carefully controlled irradiation systems, flow rates, and residence times to achieve consistent reaction performance.^73^ Engineering issues related to reactor fouling, optimizing photon flux, and ensuring an even distribution of light across longer production runs make addressing these challenges crucial for an engineer’s consideration. Therefore, the use of batch or continuous photochemical processes must be determined by the production scale, process complexity, safety considerations, and manufacturing goals.

### 3.5 Levetiracetam

Levetiracetam (**6**) belongs to the γ-butyrolactam class of AEDs and acts primarily by modulating the synaptic vesicle protein SV2A.^74^ Traditional synthetic methods for levetiracetam generally rely on chiral pool strategies, stereoselective synthesis, or resolution approaches, which often involve multiple steps and limited stereocontrol.^75^ Therefore, the development of efficient stereoselective synthetic routes under mild and sustainable conditions remains highly desirable.

To address these challenges, Colombo and colleagues reported an enantioselective organophotocatalytic strategy for the α-functionalization of aldehydes using *N*-lactam radicals, which was further applied to the telescoped continuous-flow synthesis of (*S*)-levetiracetam.^76^ The authors developed a visible-light-mediated organophotocatalytic method in which *N*-lactam radicals are generated under photoredox conditions and subsequently added to aldehydes through an enamine intermediate formed with a chiral imidazolidinone organocatalyst. The reaction between butanal and an *N*-lactam radical precursor, in the presence of the organic photocatalyst, 4-CzIPN, basic additives, and the organocatalyst under blue-light irradiation, produced the desired α-functionalized products in good yields and enantioselectivities up to 92% ee. Mechanistically, the photocatalyst generates an electrophilic N-lactam radical via single-electron transfer, which adds to the chiral enamine derived from the aldehyde and organocatalyst. Subsequent oxidation and hydrolysis regenerate the catalyst and deliver the enantioenriched α-lactam aldehyde product.

The methodology was further translated into a continuous-flow photochemical process using a Syrris Asia system equipped with 450 nm LEDs. Under optimized conditions (20 °C, 1 mol % of 4CzIPN, 30 min residence time), the reaction afforded the intermediate alcohol in 45% yield and 89% ee. Increasing the residence time to 60 min improved the yield to 60%, although the shorter residence time was selected to maximize productivity (Scheme 8).

**Scheme 8:**
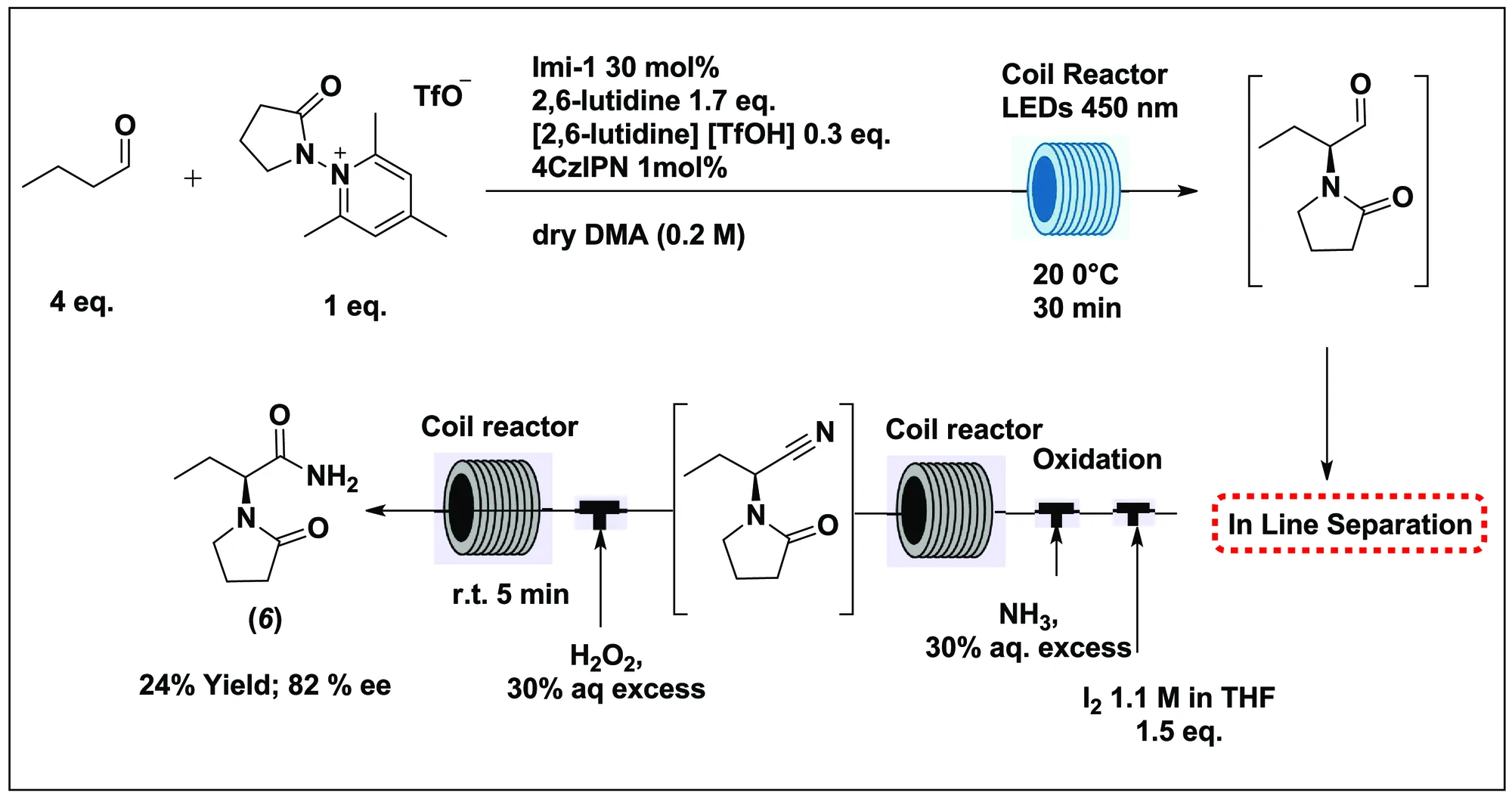
Telescoped Multistep Continuous-Flow Strategy for Synthesis of (*S*)-Levetiracetam

To demonstrate the synthetic utility of the process, the authors developed a telescoped multistep continuous-flow synthesis of (*S*)-levetiracetam. After the photochemical step, inline purification and a two-step oxidation sequence converted the intermediate aldehyde into the desired amide, ultimately yielding (*S*)-levetiracetam in 24% overall yield and 82% ee. The continuous process showed improved space-time yield and productivity compared to the batch process, highlighting the advantages of flow technology in light-driven transformations.

Overall, this work demonstrated that organophotocatalysis combined with continuous-flow processing provides an efficient platform for the sustainable synthesis of AEDs. The integration of visible-light photochemistry with flow technology enables improved reaction control, enhanced productivity, and scalable production of chiral pharmaceutical intermediates.

### 3.6 Rufinamide

The synthesis of Rufinamide (**9**), an AED used in the management of Lennox–Gastaut syndrome, has attracted significant attention due to the need for safer and more sustainable manufacturing strategies.^77^ In this context, Zhang and colleagues outlined a continuous manufacturing process for rufinamide (Scheme 9).^78^ This approach involved a cycloaddition reaction catalyzed by a copper-tubing reactor. Rufinamide was produced in an overall yield of 92% with an average residence time of about 11 min. The risks associated with safety were greatly reduced as the organoazide intermediate was not accumulated or isolated. The otherwise expensive and unstable propiolamide was produced inline and could be used without concern for polymerization or storage, and it did not require any additional functional-group modifications, since the ultimate target was the direct product.

**Scheme 9:**
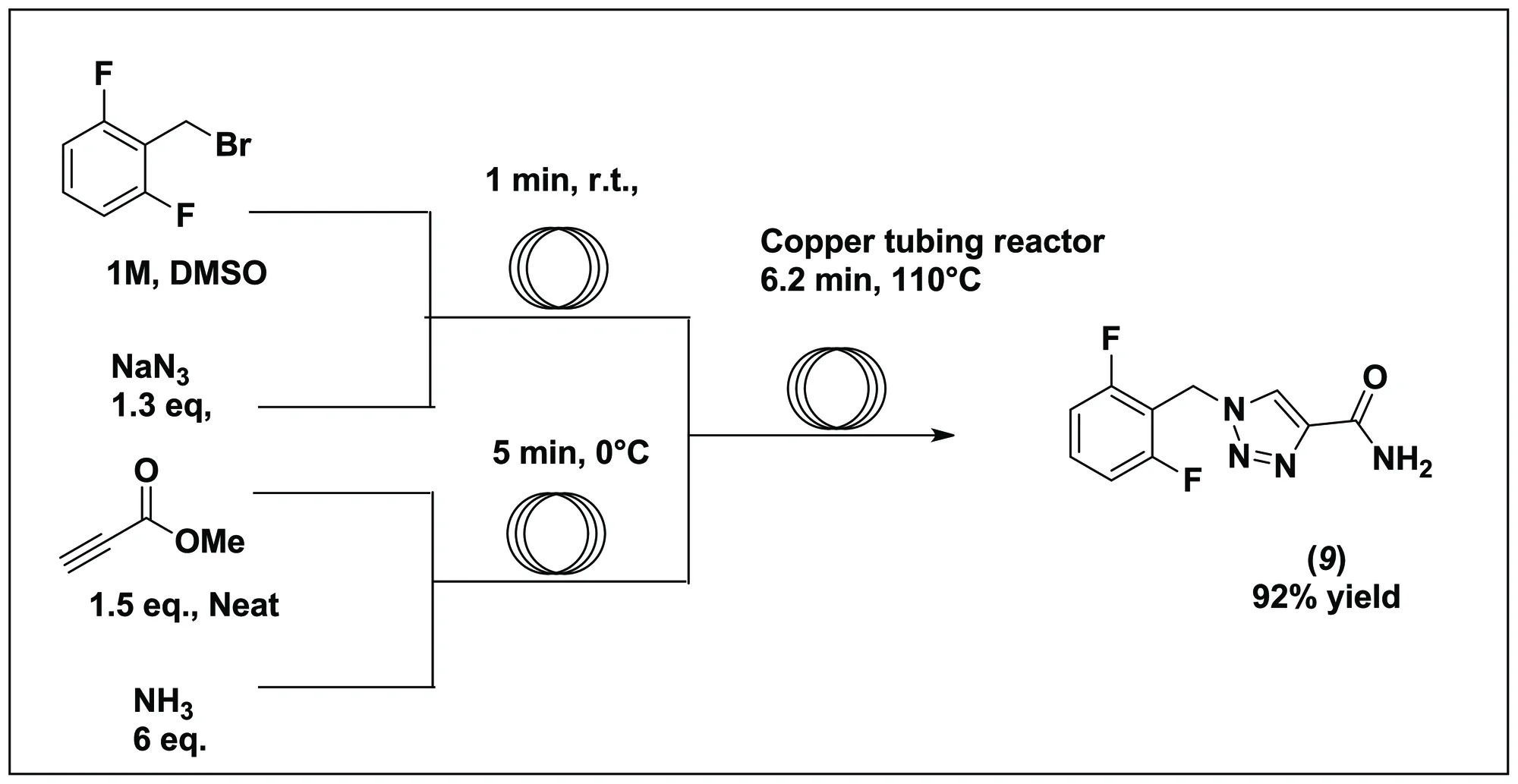
Continuous-Flow Synthesis of Rufinamide Catalysed by a Copper Tubing Reactor

Borukhova and coworkers developed an innovative multistep continuous-flow process for the synthesis of a key 1,2,3-triazole precursor of rufinamide.^79^ This approach was inspired by biological assembly-line systems, where complex molecules are synthesized through sequential, integrated transformations under controlled conditions.^80^ The authors designed a five-stage microreactor network integrating reaction and separation steps into a single continuous process (Scheme 10). The synthesis begins with 2,6-difluorobenzyl alcohol, which undergoes chlorodehydroxylation using hydrogen chloride gas to yield the corresponding benzyl chloride. The transformation represents a greener alternative to traditional chlorination methods because the only byproduct is water. Microreactors provide the opportunity for operating under intensified (high-temperature, high-pressure) conditions, providing improved reaction rates, selectivity, and safety. The second step directly converts the benzyl chloride intermediate to 2,6-difluorobenzyl azide by nucleophilic substitution with sodium azide in a biphasic flow system. Notably, this step does not require isolation of the chlorinated intermediate, which poses health and safety hazards due to its toxicity and lachrymatory properties. As a result, this step entails less hazardous handling and simplifies the overall workflow. Additionally, the continuous-flow technique produces and consumes the potentially explosive azide intermediate in situ, in small amounts, further improving process safety. An inline, membrane-based liquid–liquid separator isolates the organic azide phase, allowing it to be directly connected to the next step without further purification. The final step involves a 1,3-dipolar cycloaddition between the azide intermediate and (*E*)-methyl 3-methoxyacrylate, the latter being a cost-effective and environmentally friendly dipolarophile. The reaction occurs under superheated continuous-flow conditions (∼210 °C), providing rapid conversion to the desired triazole ester precursor. The introduction of methanol into the continuous-flow reactor subsequently promotes inline crystallization, enabling simple product isolation by filtration. Overall, the process integrates three chemical transformations and two inline separations, achieving an 82% overall yield with a productivity of 9 g h^–1^ and a total residence time of approximately 95 min.

**Scheme 10:**
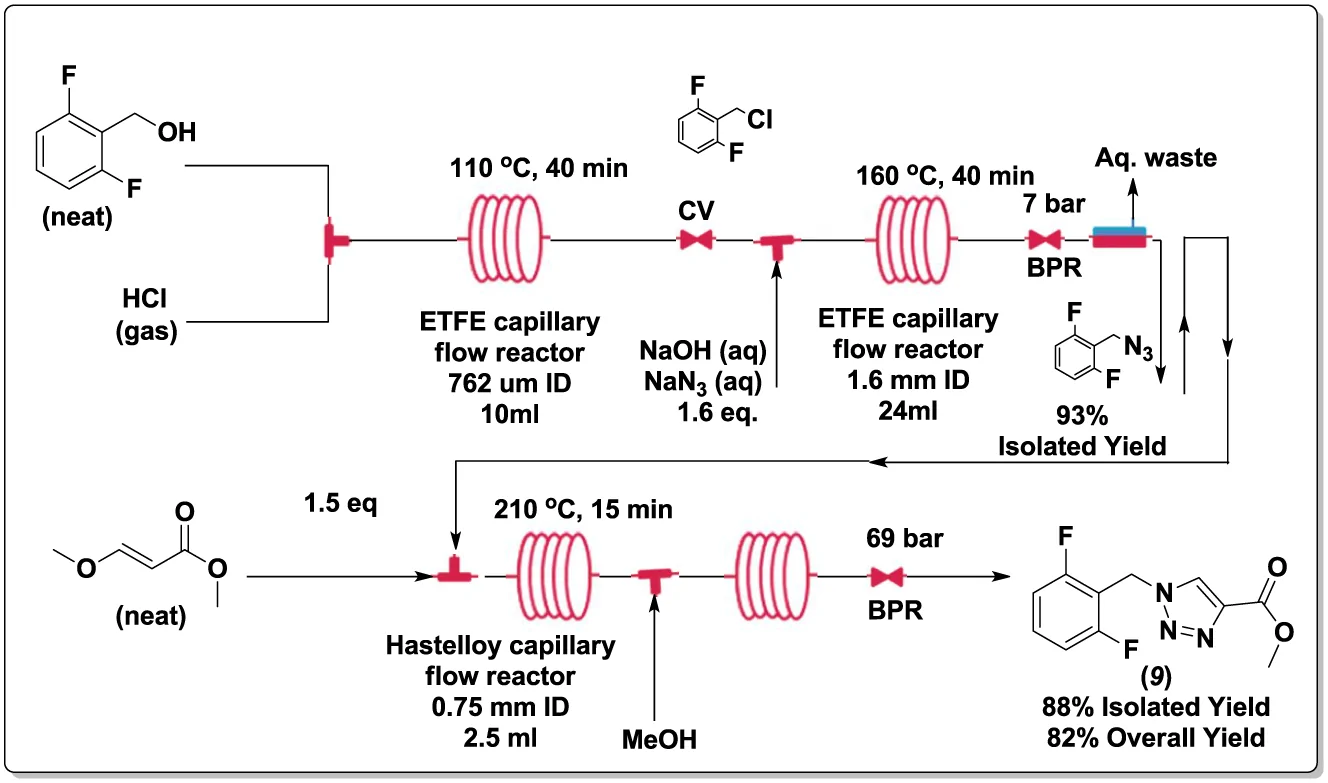
Strategy for Multi-Step Continuous-Flow Synthesis of Rufinamide Precursor

A novel continuous-flow method for synthesizing rufinamide was reported by Zhang and colleagues, combining multiple reaction steps into one and keeping the process uninterrupted by using a single solvent.^81^ This “one-flow” method involved the formation of benzyl azide (via azidation), followed by a copper-catalyzed azide-alkyne cycloaddition (CuAAC) to form the final triazole of rufinamide (Scheme 11). In order to ensure compatibility between reaction steps, compartmentalization was employed, using immobilized reagents and polymer-supported catalysts (PLs) that allowed different reaction environments to exist in the same apparatus without interfering with one another. The unique feature of this work was the use of computational solvent selection (COSMO-RS) to identify a single solvent system that supported all stages and facilitated the separation of products. The authors reported that when implemented in a microfluidic flow device, this method could potentially provide a safer route (particularly with respect to azide handling), generate less waste, and avoid the need for intermediate purification. Overall, the work showed that flow chemistry combined with intelligent reactor design and computer-aided tools can lead to a much more efficient, scalable, and environmentally friendly approach to pharmaceutical synthesis.

**Scheme 11:**
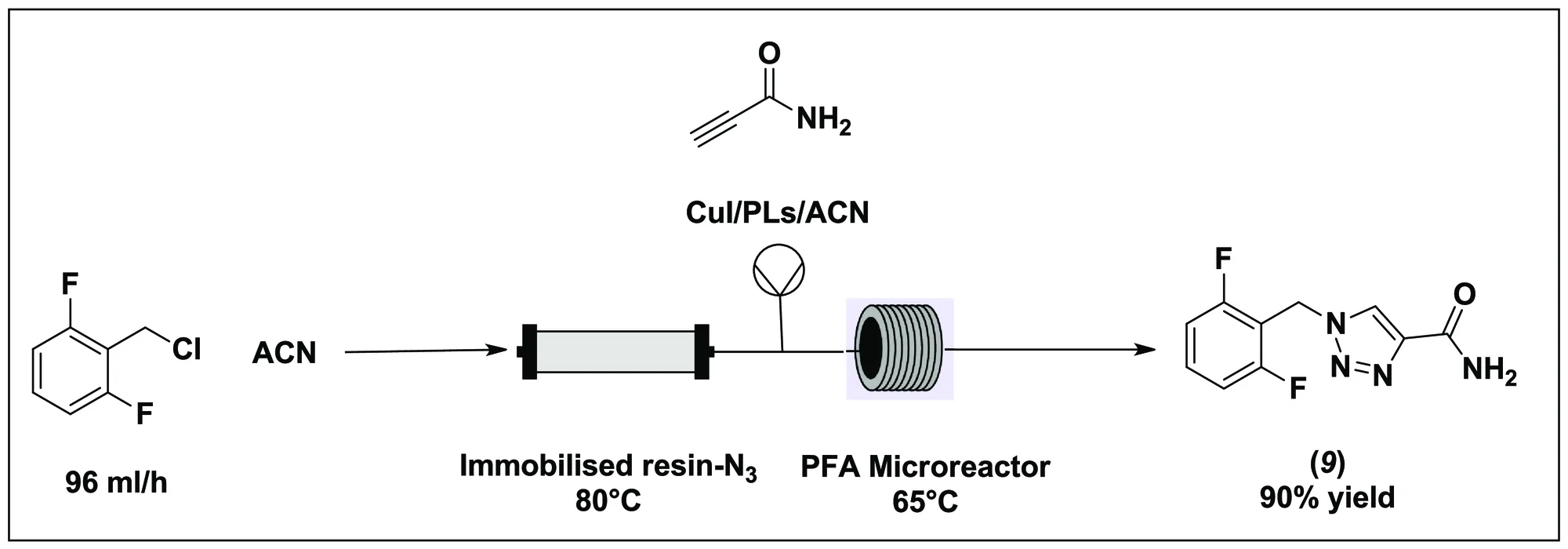
Continuous-Flow Method for Synthesizing Rufinamide

Alcalde et al. reported a flow-reactor synthesis and purification strategy for the anticonvulsant rufinamide using supercritical carbon dioxide (scCO2) (Scheme 12).^82^ The flow-reactor approach was used to synthesize triazoles, common precursors in drug discovery, via copper-catalyzed 1,3-dipolar azide/alkyne cycloaddition in scCO2, and achieved a high reaction rate of 594 g L^–1^ h^–1^. The yield for rufinamide was 52%. Another important feature of the flow reactor is that it is equipped with a chromatography column; the combination of these two devices allows for inline purification and easy analysis by HPLC of collected fractions. This continuous-flow approach had advantages over conventional batch methods, such as shorter reaction times, no need for catalysts, decreased solvent consumption, improved operator safety, and reduced complexity of downstream processing. Furthermore, this method illustrated the principles of green chemistry and process intensification, including waste minimization, energy efficiency, and combining reaction steps with separation.

**Scheme 12:**
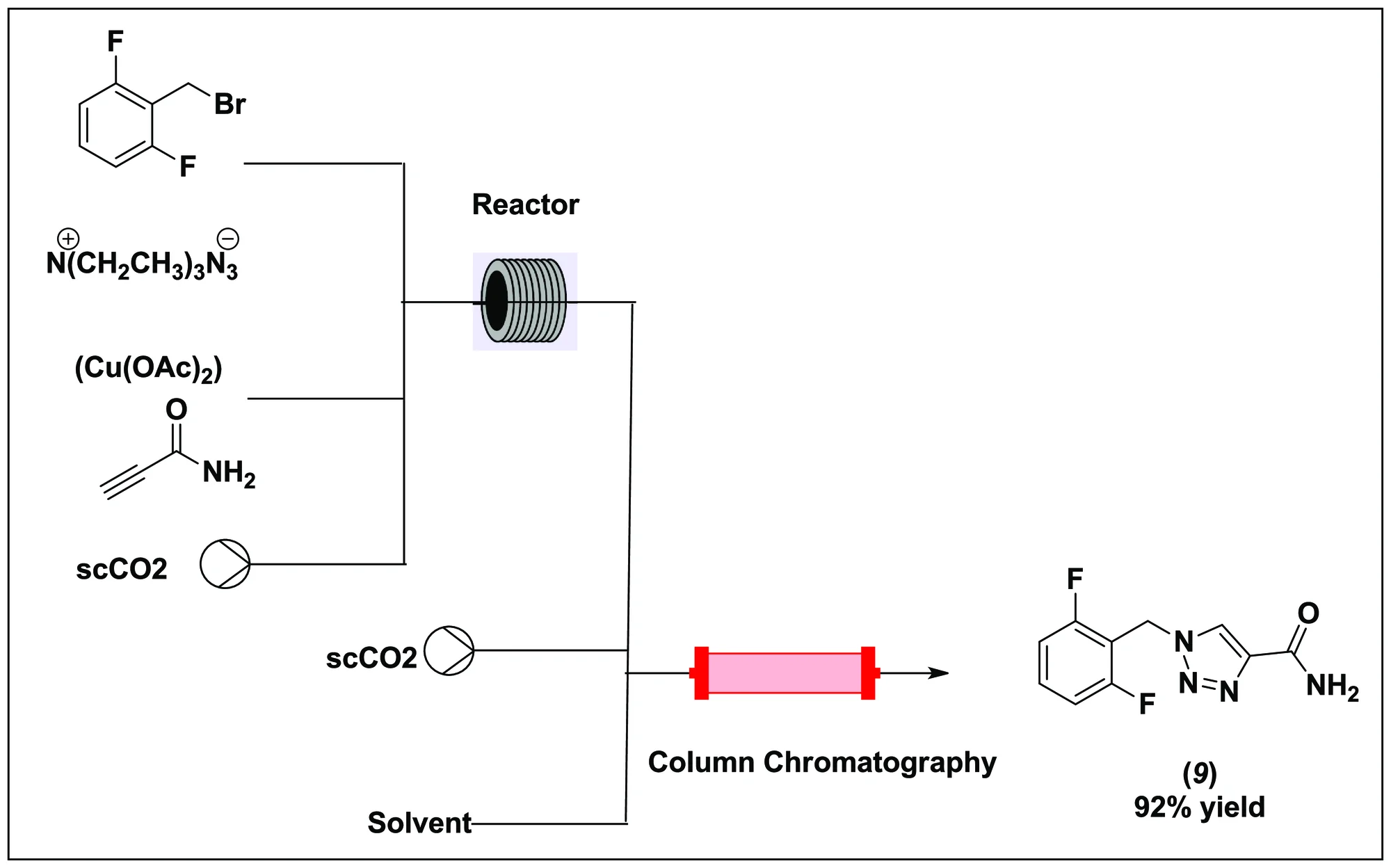
Synthesis of Rufinamide Using Supercritical Carbon Dioxide

Yuan and colleagues investigated a new dual-stage adjustable-robust system to determine ideal continuous manufacturing modes for rufinamide using mixed-integer nonlinear programming and a set of over 500 potential synthesis pathways with uncertain parameters.^83^ Based on various uncertainties, such as conversion rates, energy consumption, and production volumes, they integrated the identification of optimal reaction paths with operational decision-making, including the selection of microreactors and their operating conditions. They created an adaptive multicolumn and constraint-generation algorithm, which addresses the computational difficulties associated with mixed-integer variables by providing a more accurate and cost-effective solution to problems that are beyond the capabilities of existing methods. The results of the study show that processes designed in response to uncertainty were more reliable and cost-effective than deterministic optimization, resulting in savings of up to ∼3%.

### 3.7 Comparative Assessment of Continuous-Flow Reactor Platforms

As demonstrated previously, there is no universally optimal reactor type for the manufacture of AEDs via continuous-flow synthesis. The reactor selected by the manufacturer will depend on several factors, including the type of chemical transformation, the catalyst used, the reaction kinetics, heat and mass transfer properties, safety considerations, and the degree of integration required to achieve the desired outcome. Each previously discussed study evaluated specific synthetic pathways, but through comparison of the reactor platforms used across studies, commonalities and contrasts in their capabilities, limitations, and applications in pharmaceutical production became evident. Therefore, Table 3 summarizes the major continuous-flow reactor platforms used in the manufacture of AEDs, including representative applications, operational advantages and disadvantages, typical processing characteristics, and relevance to green and sustainable manufacturing. This comparison should help the reader understand the reasons for selecting the reactor class used for a particular continuous-flow transformation.

**Table 3:** Comparison of Mainstream Continuous-Flow Reactor Platforms Used in Pharmaceutical Synthesis and Their Relevance to AED Synthesis

Rector platform	Application	Representative AED example in this review	Advantage	Limitation	Green chemistry consideration
**Packed bed reactor**	Heterogeneous catalytic reactions, asymmetric catalysis	Pregabalin	Catalyst immobilization, easy catalyst recovery, long-term continuous operation, telescoping	Catalyst deactivation, pressure drop, bed fouling	Reduced catalyst waste, catalyst reuse, lower solvent consumption
**Tubular reactor**	Fast homogeneous reactions, multistep synthesis	Sodium valproate, Diazepam	Excellent heat and mass transfer, simple scale-up, good temperature control	Mixing limitations for multiphasic systems	Lower reaction inventory, improved safety, lower waste generation
**Continuous photoflow reactor**	Photochemical and photocatalytic transformations	Brivaracetam, Levetiracetam	Efficient light penetration; short irradiation time, excellent temperature control	Scale-up requires uniform photon distribution, specialized light source	Improved energy utilization, reduced reaction time
**Copper tubing reactor**	Click chemistry, hazardous intermediate handling	Rufinamide	Safe in situ azide handling, catalytic reactor walls, simple construction	Limited reaction scope, metal compatibility	Safer handling of energetic intermediates
**Integrated microreactors**	Multistep continuous synthesis with separations	Rufinamide	Process intensification, inline extraction, telescoping	Complex equipment and control strategy	Reduced solvent use, fewer isolation steps
**Supercritical flow reactors**	Continuous synthesis coupled with purification	Rufinamide	Integrated synthesis and purification, reduced organic solvent use	High-pressure operation, specialized equipment	Green solvent, simplified downstream processing

According to the data in Table 2, the advantages and limitations of different reactor designs depend on the types of reactions required for each platform. For example, packed-bed reactors are advantageous for heterogeneous catalyst reactions because of the issues associated with immobilizing catalysts, recovering them, reducing product contamination by metals, and enabling long-term continuous use of the catalyst.^84,85^ Accordingly, these reactor styles have been successfully utilized in the asymmetric catalytic synthesis of pregabalin. Another versatile reactor style used for homogeneous reactions and telescoped multistep processes is the continuous tubular reactor. Continuous tubular reactors have excellent heat and mass transfer characteristics, making them highly scalable either by increasing throughput or by numbering up.^86^ These advantages make continuous tubular reactors excellent options for multistep manufacturing processes, as evidenced by the synthesis of sodium valproate and diazepam. Photochemical flow reactors significantly increase the efficiency of visible-light-mediated transformations compared with conventional batch photochemical reactions and require much less time for asymmetric synthesis.^87^ In addition, some specialized reactor configurations, such as copper tubing reactors, offer a significant safety advantage by reducing the amount of hazardous intermediates.^88^ Integrated microreactor networks represent another example of a specialized reactor style that can combine multiple reaction/separation steps into a single, continuous manufacturing process.^89^ Similarly, supercritical CO_2_-flow reactors exemplify this trend of combining reaction and purification into continuous processes while reducing the amount of organic solvent consumed.^82^ As noted earlier, the reactor configuration should be determined by factors such as reaction efficiency, catalyst compatibility and performance, safety, scalability, downstream processing requirements, and sustainability goals.

## 4 Practical Considerations for Scale-Up and Industrial Translation

Despite the significant improvements in reaction efficiency, safety, and process intensification, numerous engineering and operational challenges must be solved to successfully transition laboratory-scale demonstrations of continuous-flow synthesis discussed throughout this review into large-scale production for industrial use. To realize the industrial potential of continuous-flow synthesis of AEDs, it is critical to evaluate processes for scalability, assess long-term operational reliability, and understand the integration of downstream processing, process monitoring, and regulatory considerations (Table 4).

**Table 4:** Practical Considerations for Industrial Translation of the Reported Continuous-Flow Syntheses of AEDs

AED	Performance reported	Pretreatment consideration	Downstream processing	Practical challenges	Future development opportunities
**Pregabalin**	Stable continuous telescoped synthesis	Preparation of catalyst-packed reactor and controlled reagent feed	Product isolation after telescoped synthesis	Long-term catalyst stability, catalyst replacement, pressure drop in packed-bed reactors	Use more durable immobilized catalysts and apply PAT to monitor the catalysts.
**Sodium valproate**	Integrated three-step continuous synthesis	Continuous reagent delivery, controlled hydrolysis	Inline extraction, product isolation	Integration of downstream purification	Fully continuous workup and crystallization
**Diazepam**	Rapid telescoped multistep synthesis	Accurate reagent metering and temperature control	Recrystallization to obtain a high-purity product	Integration of purification with telescoped synthesis	End-to-end continuous manufacturing and automated optimization
**Brivaracetam**	Photochemical Synthesis	Preparation of photocatalyst, Controlled irradiation	Isolation of final product	Photoreactor productivity, Uniform light distribution during scale-up	Numbering-up photoreactors, inline stereochemical monitoring
**Levetiracetam**	Multistep photochemical Synthesis	Controlled reagent addition	Product isolation	Further improvement of overall process efficiency and productivity	AI-assisted process optimization and PAT integration
**Rufinamide**	Azide chemistry, Integrated reaction-separation	Controlled generation of azide, Safe handling	Inline separation, chromatographic purification	Long-term reactor stability, continuous handling of hazardous intermediates	Continuous manufacturing with inline purification and real-time monitoring

### 4.1 Considerations for Developing Scalable Continuous-Flow Manufacturing

Unlike conventional batch processes, increasing the scale of continuous-flow chemistry does not necessarily require increasing the reactor volume. There are multiple ways to increase production, including increasing the time the equipment is used, increasing the rate at which material is processed while maintaining the equipment’s specific performance, or using a “numbering-up” approach, in which multiple identical channels within a reactor are run simultaneously. Most of these methods retain many of the highly desirable heat and mass transfer features observed in microstructured reactors, while greatly reducing the risks traditionally associated with geometric scale-up.^90^

The ability to operate consistently over long periods is one of the many advantages of continuous-flow processes. However, one of the key requirements for commercial-scale implementation of continuous-flow manufacturing is ensuring that all systems remain operational for extended periods without interruption, while maintaining consistent product quality and reproducible processes. As production capacities are increased, the need to increase the throughput of reactors, provide adequate and uniform mixing of materials during production in larger-scale systems, and eliminate pressure fluctuations along the length of the reactor becomes more pressing. Similarly, heterogeneous catalysts require thorough consideration of catalyst longevity, poisoning, leaching, cracking, fouling, and replacement strategies with extended use.^91^ Additionally, significant technological issues, such as channel blocking, particle accumulation, and inefficient heat and mass transfer, continue to hamper further development of continuous-flow processes for industrial applications. For long-term manufacturing processes, pump reliability, pressure stability, solvent compatibility, and the durability of reactor materials also need to be evaluated.^92^ These problems are a cause for concern in pharmaceutical manufacturing because of the stringent quality demands. Consequently, future reactor designs will need to focus on increased catalyst durability, creation of fouling-resistant designs, use of predictive maintenance strategies, and development of robust process control systems capable of maintaining steady-state operation throughout long-term manufacturing campaigns.

### 4.2 Downstream Processing Integration

In traditional pharmaceutical manufacturing, many times, isolation, purification, solvent exchange, and crystallization need to be performed separately after every reaction. This results in significant waste generation, solvent usage, and time lost in the manufacturing cycle. The goal of continuous manufacturing is to reduce the number of times these discontinuities occur by integrating the reaction and purification into a single process. The review presents several examples illustrating the advantages of incorporating downstream integration. As discussed earlier, incorporating inline extractions into the sodium valproate process enabled simplified product isolation while yielding high-purity products with favorable green metrics. Similarly, in the case of rufinamide, an integrated microreactor platform was developed to consolidate three reaction steps into a single process with continuous liquid–liquid separation, thereby eliminating manual handling and improving process safety. The implementation of continuous chromatographic purification as part of the supercritical CO_2_ process also demonstrates the trend toward combining synthesis and purification on a single manufacturing platform.

Despite these advancements, the continued integration of downstream processing remains a significant bottleneck for pharmaceutical continuous manufacturing. Continuous crystallization, solvent recycling, filtration and drying, and formulation operations all require coordination to work together with upstream reaction throughput.^19^ There is a risk that any disturbance during downstream operations will propagate back through the entire manufacturing process if it is not properly controlled. Therefore, there is a need for future research on the development of end-to-end manufacturing systems that are fully integrated.

### 4.3 Process Analytical Technology (PAT), Automation, and AI-Assisted Optimization

PAT has enabled advances in ongoing pharmaceutical production through contemporary technological options.^93^ By utilizing real-time analytical capabilities, PAT enables the monitoring of critical process parameters during synthesis and the making of required adjustments. This ensures consistently high-quality products. Recent innovations, including inline spectroscopy, chromatography, reaction calorimetry, particle imaging, and data analysis, provide continuous monitoring of reaction progress, conversion rates, and impurity levels in flow reaction processes. With the development of new phase-change and phase-separation chemical manufacturing technologies, as well as digital twins and artificial intelligence, process capabilities will continue to improve yield, production accuracy, reliability, predictive maintenance, fault detection, and design flexibility in ongoing production operations.

### 4.4 Industrial and Regulatory Viewpoints

Increasingly, regulatory agencies recognize the potential of continuous manufacturing as a viable alternative to batch manufacturing for improving process consistency, enhancing product quality, and strengthening supply chain resilience. Regulatory agencies, including the FDA, EMA, and PMDA, have confirmed that continuous manufacturing aligns with existing frameworks such as ICH Q8, Q9, and Q10.^15^ Additionally, the International Council for Harmonization (ICH) has published ICH Q13, which offers specific guidance on continuous manufacturing.^94^ In 2019, the U.S. FDA released a draft guidance titled “Quality Considerations for Continuous Manufacturing: Guidance for Industry”.^95^ This document outlines quality system considerations for continuous manufacturing, including control strategies and process validation. Similarly, case studies conducted by pharmaceutical companies indicate growing industry interest in continuous synthesis and separation processes, as well as integrated end-to-end API manufacturing. However, several barriers still limit broader implementation in practice. For example, initial investment in capital equipment, process validation, workforce training, integration of multiple unit operations, and demonstrating the long-term manufacturing robustness of a new technology are all substantial considerations as the technology is adopted. Therefore, developing standardized reactor platforms and harmonizing regulatory pathways to support the implementation of continuous-flow technologies in pharmaceutical manufacturing will be critical facilitators of their industrial adoption.

## 5 Toward Failure-Resistant Flow Chemistry: Outlook

There have been many examples throughout this review that demonstrate how continuous-flow chemistry has transitioned from an enabling technology for synthetic manufacturing to a viable method for large-scale production of several AEDs. On the basis of the case studies summarized in this review, the following areas of research will be critical for increasing the use of continuous-flow synthesis for the manufacture of AEDs.

### 5.1 Development of Telescoped Multistep Flow Synthesis

On the basis of the examples reported in this review, telescoped multimolecular flow synthesis is among the more promising methods for improving manufacturing efficiency with continuous-flow processes. For instance, the synthesis of diazepam and sodium valproate demonstrates that incorporating sequential reactions into a single continuous-flow process reduces the need for intermediate isolation and the time required to carry out multiple reactions, lowers solvent consumption, and increases overall process efficiency. Therefore, future research will focus on the number of additional reactions and purification steps that are integrated, as well as compatibility among different transformations. In addition, fully continuous-flow production platforms must include inline extraction, crystallization, and purification stages, which would further reduce downstream processing requirements and improve the environmental sustainability and operational flexibility of the overall flow manufacturing process.^89^

### 5.2 Enhancing Stability for Hazardous Intermediates

Rufinamide’s production through a continuous-flow process showcases one of the key benefits of flow chemistry-creating reactive azide intermediates safely. While studies have provided evidence of safe process design, future work must continue to focus on improving the overall operational stability of flow systems during the continuous production of pharmaceutical products. The engineering challenges necessary to support continued production using continuous-flow systems need to be addressed through further study, as reactor fouling, pressure fluctuations, catalyst life, and the integration of continuous downstream purification technologies into flow systems remain essential considerations. Additionally, the broader implementation of inline monitoring and PAT will enable real-time detection of deviations from process performance criteria and improve manufacturing reliability for reactions involving hazardous intermediates.

### 5.3 Chiral Photoflow Methods for AED Synthesis

The success of producing brivaracetam and levetiracetam *via* continuous photochemical processes demonstrates the capability of photoflow chemistry to manufacture complex, chiral AEDs. The use of continuous photoflow reactors results in improved photon usage, better temperature control, and significantly shorter reaction times than conventional batch methods, while maintaining high levels of stereochemical purity.^96^ Future research into asymmetric photoredox catalysis to develop additional chiral AEDs is warranted, along with continued efforts to develop more efficient photocatalysts, to increase photoreactor productivity, and to develop scalable “numbering-up” approaches for the industrial production of chiral AEDs. The incorporation of inline analysis of stereochemical properties would enable real-time optimization of flow-type processes that rely on enantioselectivity.

## 6 Conclusion

As a manufacturing method for AEDs, continuous-flow chemistry is a mature and established approach that offers significant advantages over conventional batch processing with regard to (1) reaction safety, (2) process intensification, (3) heat and mass transfer, (4) scalability, and (5) sustainability. The examples discussed in this paper show that various continuous-flow strategies have been successfully utilized to synthesize AEDs, including asymmetric catalysis, telescoped multistep synthesis, photochemistry, and the safe use of hazardous intermediates. These developments have led to reduced reaction times, higher product yields, increased selectivity, reduced solvent consumption, and reduced waste generation.

However, these achievements will require continued advancement of continuous-flow chemistry to enable broader industrial adoption in the manufacturing of AEDs, particularly to improve long-term process robustness, reactor scalability, catalyst stability, and the integration of continuous downstream processing. Future research should continue to focus on multistep telescoped syntheses for AEDs, establishing more robust flow systems for hazardous and highly reactive intermediates, and expanding the application of asymmetric photoflow syntheses for the manufacture of chiral AEDs. In addition, continued innovation in integrating process analytical technologies, automation, and artificial intelligence-driven optimization is expected to accelerate the development of robust, end-to-end continuous manufacturing platforms.

To summarize, the studies presented in this review suggest that continuous-flow synthesis technology is being developed for AED synthesis, moving beyond laboratory synthesis. Synthetic chemists, chemical engineers, and pharmaceutical scientists must continue their collaborative efforts to translate emerging laboratory technologies into reliable, cost-effective, and sustainable manufacturing methods for AEDs that meet future clinical and industrial needs.
